# Social Features of Online Networks: The Strength of Intermediary Ties in Online Social Media

**DOI:** 10.1371/journal.pone.0029358

**Published:** 2012-01-11

**Authors:** Przemyslaw A. Grabowicz, José J. Ramasco, Esteban Moro, Josep M. Pujol, Victor M. Eguiluz

**Affiliations:** 1 Instituto de Fisica Interdisciplinaria y Sistemas Complejos (CSIC-UIB), Palma de Mallorca, Spain; 2 Instituto de Ingeniera del Conocimiento, Universidad Autónoma de Madrid, Madrid, Spain; 3 Instituto de Ciencias Matemáticas CSIC-UAM-UC3M-UCM, Departamento de Matemáticas y GISC, Universidad Carlos III de Madrid, Leganés, Spain; 4 Telefónica Research, Barcelona, Spain; 5 3scale Networks, Barcelona, Spain; University of Zaragoza, Spain

## Abstract

An increasing fraction of today's social interactions occur using online social media as communication channels. Recent worldwide events, such as social movements in Spain or revolts in the Middle East, highlight their capacity to boost people's coordination. Online networks display in general a rich internal structure where users can choose among different types and intensity of interactions. Despite this, there are still open questions regarding the social value of online interactions. For example, the existence of users with millions of online friends sheds doubts on the relevance of these relations. In this work, we focus on Twitter, one of the most popular online social networks, and find that the network formed by the basic type of connections is organized in groups. The activity of the users conforms to the landscape determined by such groups. Furthermore, Twitter's distinction between different types of interactions allows us to establish a parallelism between online and offline social networks: personal interactions are more likely to occur on internal links to the groups (the weakness of strong ties); events transmitting new information go preferentially through links connecting different groups (the strength of weak ties) or even more through links connecting to users belonging to several groups that act as brokers (the strength of intermediary ties).

## Introduction

There exists an open discussion on the validity of online interactions as indicators of real social activity [1]–[6]. Most of the online social networks incorporate several types of user-user interactions that satisfy the need for different level of involvement or relation intensity between users [7]–[11]. The cost of establishing the cheapest relation is usually very low, and it requires the acceptation or simply the notification to the targeted user. These connections can accumulate due to the asymmetric social cost of cutting and creating them, and pile up to the astronomic numbers that capture popular imagination [3]. If the number of connections increases to the thousands or the millions, the amount of effort that a user can invest into the relation that each link represents must fall to near zero. Does this mean that online networks are irrelevant for understanding social relations, or for predicting where higher quality activity (e.g., personal communications, information transmission events) is taking place? By analyzing the clusters of the network formed by the cheapest connections between users of Twitter, we show that even this network bears valuable information on the localization of more personal interactions between users. Furthermore, we are able to identify some users that act as brokers of information between groups.

The theory known as *the strength of weak ties* proposed by Granovetter [12] deals with the relation between structure, intensity of social ties and diffusion of information in offline social networks. It has raised some interest in the last decades [12]–[15] and its predictions have been checked in a mobile phone calls dataset [14]. On one hand, a tie can be characterized by its strength, which is related to the time spend together, intimacy and emotional intensity of a relation. Strong ties refer to relations with close friends or relatives, while weak ties represent links with distant acquaintances. On the other hand, a tie can be characterized by its position in the network. Social networks are usually composed of groups of close connected individuals, called communities, connected among them by long range ties known as bridges. A tie can thus be internal to a group or a bridge. Grannoveter's theory predicts that weak ties act as bridges between groups and are important for the diffusion of new information across the network, while strong ties are usually located at the interior of the groups. Burt's work [16] later emphasizes the advantage of connecting different groups (bridging structural holes) to access novel information due to the diversity in the sources. More recent works, however, point out that information propagation may be dependent on the type of content transmitted [17], [18] and on a *diversity-bandwidth tradeoff* [19]. The bandwidth of a tie is defined as the rate of information transmission per unit of time. Aral et al. [19] note that weak ties interact infrequently, therefore have low bandwidth, whereas strong ties interact more often and have high bandwidth. The authors claim that both diversity and bandwidth are relevant for the diffusion of novel information. Since both are anticorrelated, there has to be a tradeoff to reach an optimal point in the propagation of new information. They also suggest that strong ties may be important to propagate information depending on the structural diversity, the number of topics and the dynamic of the information. Due to the different nature of online and offline interactions, it is not clear whether online networks organize following the previous principles. Our aim in this work is to test if these theories apply also to online social networks.

Online networks are promising for such studies because of the wide data availability and the fact that different type of interactions are explicitly separated: e.g., information diffusion events are distinguished from more personal communications. Diffusion events are implemented as a system option in the form of *share* or *repost* buttons with which it is enough to single-click on a piece of information to rebroadcast it to all the users' contacts. This is in contrast to personal communications and information creation for which more effort has to be invested to write a short message and (for personal communication) to select the recipient. All these features are present in Twitter, which is a micro-blogging social site. The users, identified with a username, can write short messages of up to 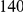 characters (tweets) that are then broadcasted to their followers. When a new follower relation is established, the targeted user is notified although his or her explicit permission is not required. This is the basic type of relation in the system [20]–[22], which generates a directed graph connecting the users: the follower network. After some time of functioning, some peculiar behaviors started to extend among Twitter users leading to the emergence of particular types of interactions. These different types of interactions have been later implemented as part of Twitter's system [23]. *Mentions* (tweets containing @username) are messages which are either directed only to the corresponding user or mentioning the targeted user as relevant to the information expressed to a broader audience. A *retweet* (RT @username) corresponds to content forward with the specified user as the nominal source. In contrast to the normal tweets, mentions usually include personal conversations or references [8] while retweets are highly relevant for the viral propagation of information [24]. This particular distinction between different types of interactions qualifies Twitter as a perfect system to analyze the relation between topology, strength of social relation and information diffusion in online social networks.

The properties of the follower network have been extensively analyzed especially in relation to its topological structure, propagation of information, homophily, tie formation and decay, etc [25]–[31]. Finding users with thousands or even millions of followers is not exceptional [3], so the question is whether the structure of the follower network carries any information on where personal relations (mentions) or information transmission events (retweets) take place. To answer this question, we first analyze a sample of the follower network with clustering-detection algorithms and identify a set of groups. Our dataset is a sample of the network containing 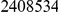 users connected with 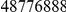 follower relations, as well as the tweets, retweets, mentions, and was gathered through the Twitter API during November and December of 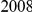 [30], [32], [33] (see the Methods Section for further detail). Whether the clusters we identify are traces of underlying social groups (online or offline) is a question we cannot answer with the available information. We follow an alternative path by checking the correlation between the location of the personal conversations (mentions) and information diffusion events (retweets) and the structural properties of the link bearing those activities with respect to the detected groups in the network. Note that we consider mentions and retweets to happen always on follower links. This allow us to describe user activity in terms of the detected groups.

## Results

### 2.1 Description of the groups

**Figure 1:**
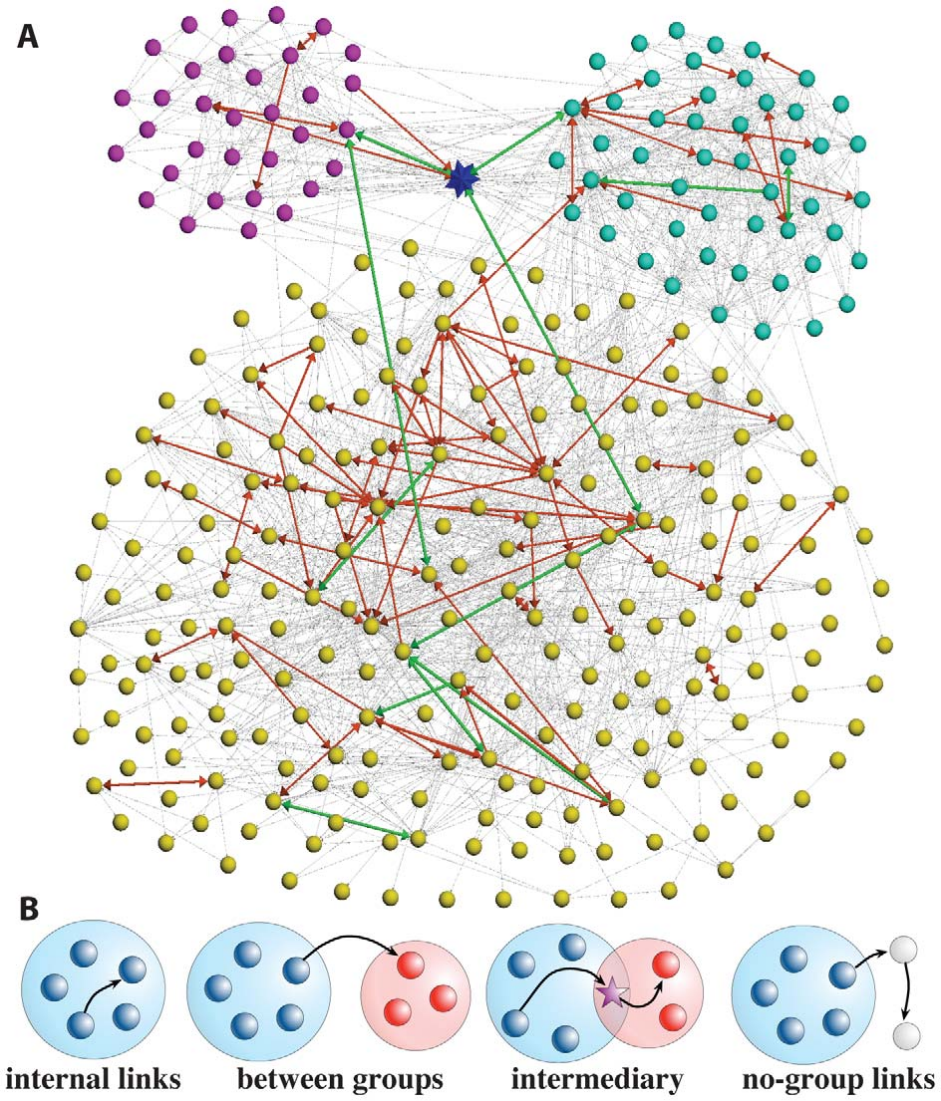
Groups and links. (A) Sample of Twitter network: nodes represent users and links, interactions. The follower connections are plotted as gray arrows, mentions in red, and retweets in green. The width of the arrows is proportional to the number of times that the link has been used for mentions. We display three groups (yellow, purple and turquoise) and a user (blue star) belonging to two groups. (B) Different types of links depending on their position with respect to the groups' structure: internal, between groups, intermediary links and no-group links.

Our first step is to identify the groups in the follower network. Clustering in large graphs is still a topic of very active research and many algorithms are available [34]. Due to the size, density, and directness of the follower network and in order to capture the possible inclusion of users in multiple groups or in none, we have used Oslom [35], [36] (see Methods). The analysis has also been performed with other clustering techniques [37]–[41], reaching similar conclusions (see Figs. S6, S7, S8, S9, S10, S11, S12, S13, S14 and Table S1] for a detailed account on these results). We have detected 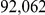 groups, three of which are graphically depicted in Figure 1A with each sphere corresponding to a single user. In general, the links can be classified according to their position with respect to the user groups: internal, between groups, intermediary and links involving nodes not assigned to any group as shown in Figure 1B.

**Figure 2:**
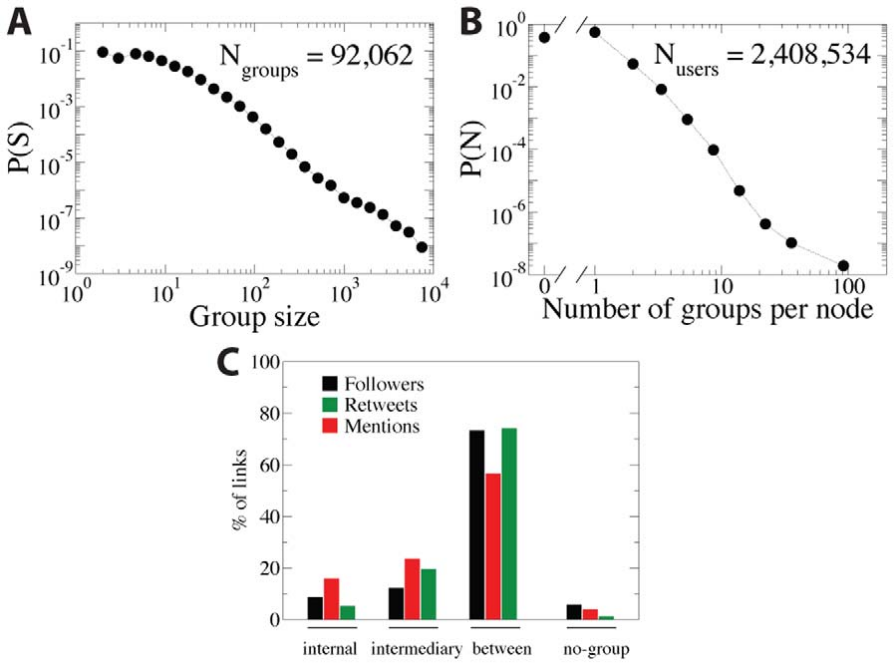
Group and link statistics. (A) Size distribution of the group. (B) Distribution of the number of groups to which each user is assigned. (C) Percentage of links of different types, e.g. follower links (black bars), links with mentions (red bars) or retweets (green bars), staying in particular topological localizations in respect to detected groups.

The statistics characterizing the groups and links are displayed in Figure 2. The group size distribution decays slowly for three orders of magnitude and does not show a characteristic group size (Figure 2A). For instance, the largest group contains around 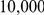 users. Also the number of groups each user belongs to shows high heterogeneity: 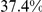 of the users has not been allocated to any group, while there exists a user belonging to more than 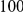 groups (see Figure 2B). The percentage of links falling in the different types regarding the groups is depicted in Figure 2C. Although the non-classified users are 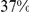 of the total, the links connected to them are less than 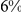 and the percentage is even lower for those with mentions or retweets. The most common type of connections is the between-group links. One may wonder if the algorithm for clusters detection is doing a good job when there is such a large proportion of between-group links. The clustering method is trying to find groups of mutually interconnected nodes that would be extremely rare in a randomized instance of the network, rather than optimizing the ratio between number of between-group and internal links. In 1, S2, S3, S4, S5, this argument is further developed and the capacity of Oslom to detect planted communities is proved in a benchmark even in situations with a high ratio between the number of between-groups and internal links. Another relevant point to highlight is the different potential of each type of links to carry mentions and retweets. As it can be seen in the Figure 2C, the red bars for mentions in internal links and intermediary links almost double the abundance of links in the follower network in these categories. The links between groups, on the other hand, attract far less mentions.

### 2.2 The strength of ties

Besides their location with respect to the groups, the links can be also characterized by their intensity. In Twitter mentions are typically used for personal communication, which establishes a parallelism between links with mentions and strength of social ties. The more mentions has been exchanged between two users, even more so if reciprocated, the stronger we consider the tie between them. We define intensity of a link as the number of mentions interchanged on it. Different predictors have been considered to estimate social tie strength [42] including, for instance, time spent together [42] or the duration of phone calls [14]. We consider the intensity as an approximation to social strength given that writing a mention involves some effort and addresses only single targeted users.

### 2.3 Internal links

**Figure 3:**
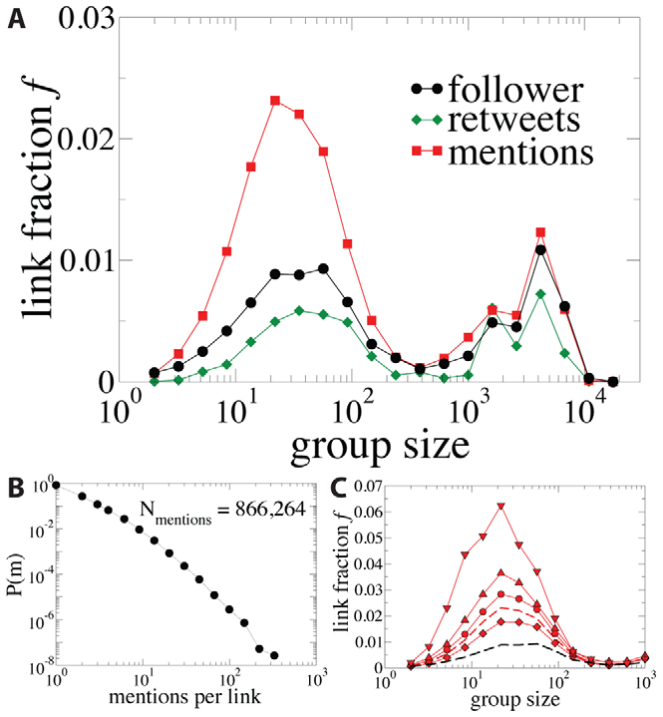
Internal activity. (A) Fraction 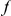 of internal links as a function of the group size in number of users. The curve for the follower network acts as baseline for mentions and retweets. Note that if mentions/retweets were randomly appearing over follower links then the red/green curve should match the black curve. (B) Distribution of the number of mentions per link. (C) Fraction of links with mentions as a function of their intensity. The dashed curves are the total for the follower network (black) and for the links with mentions (red). While the other curves correspond (from bottom to top) to fractions of links with: 1 non-reciprocated mention (diamonds), 3 mentions (circles), 6 mentions (triangle up) and more than 6 reciprocated mentions (triangle down).

According to Granovetter's theory, one could expect the internal connections inside a group to bear closer relations. Mechanisms such as homophily [43], cognitive balance [44], [45] or triadic closure [12] favor this kind of structural configurations. Unfortunately, we have no means to measure the closeness of a user-user relation in a sociological sense in our Twitter dataset. However we can verify whether the link has been used for mentions, whether the interchange has been reciprocated or whether it has happened more than once. We define the fraction 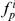 of links with interaction 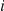 in position 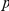 with respect to the groups of size 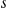 as
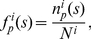
(1)where 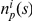 is the number of links with that type of interaction in position 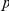 with respect to the groups of size 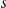 and 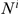 in the total number of links with interaction 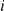. The fractions 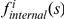 reveals an interesting pattern as function of the group size as can be seen in Figure 3A. Note that the fraction of links in the follower network (black curve) is taken as the reference for comparison. Links with mentions are more abundant as internal links than the baseline follower relations for groups of size up to 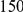 users. This particular value brings reminiscences of the quantity known as the Dunbar number [46], the cognitive limit to the number of people with whom each person can have a close relationship and that has recently been discussed in the context of Twitter [47]. Although we have identified larger groups, the density of mentions is similar to the density of links in the follower network. In addition, the distribution of the number of times that a link is used (intensity) for mentions is wide, which allows for a systematic study of the dependence of intensity and position (see Figure 3B). The more intense (or reciprocated) a link with mentions is, the more likely it becomes to find this link as internal (Figure 3C). This corresponds to Granovetter expectation that the stronger the tie is the higher number of mutual contacts of both parties it has and the higher the chance that the parties belong to the same group.

### 2.4 Links between groups

**Figure 4:**
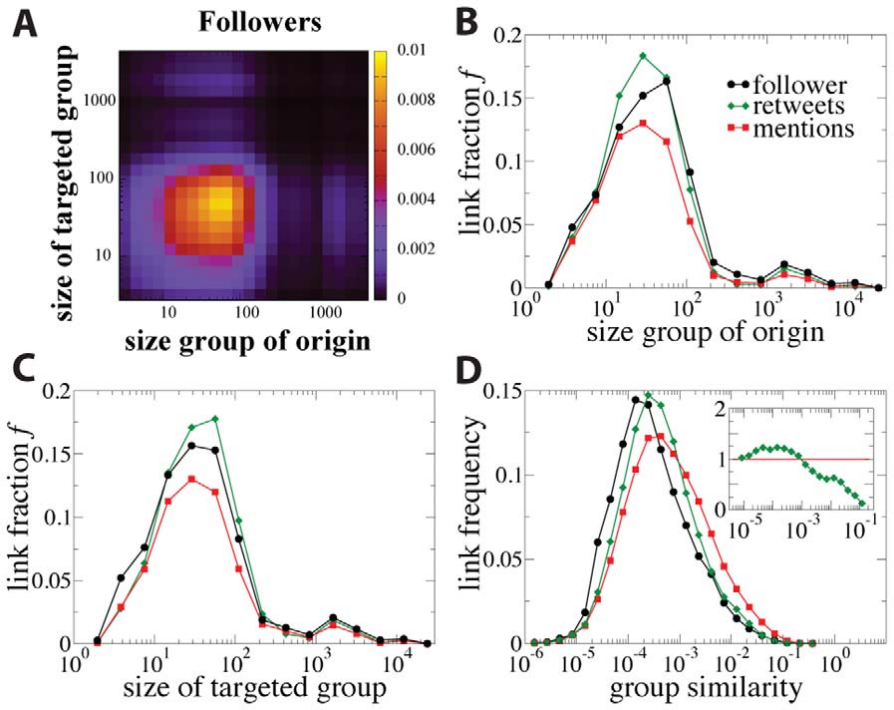
Group-group activity. (A) Distribution of the number of links in the follower network between groups as a function of the size of the groups. (B) Fractions 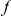 of links of the different types (follower, with mentions and with retweets) as a function of the size of the group at the link origin, and (C) at the targeted group. (D) Frequency of between-group links as a function of the group-group similarity for the different type of links. In the inset, ratio between the frequency of links with retweets and with mentions.

The next question to consider is the characteristics of links between groups. These links occur mainly between groups containing less than 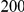 users (Figure 4A–C). However, their frequency depends on the quality of the links (if they bear mentions or retweets). While links with mentions are less abundant than the baseline, those with retweets are slightly more abundant. According to the strength of weak ties theory [12], [14]–[16], weak links are typically connections between persons not sharing neighbors, being important to keep the network connected and for information diffusion. We investigate whether the links between groups play a similar role in the online network as information transmitters. The actions more related to information diffusion are retweets [24] that show a slight preference for occurring on between-group links (Figures 4B and 4C). This preference is enhanced when the similarity between connected groups is taken into account. We define the similarity between two groups, A and B, in terms of the Jaccard index of their connections:
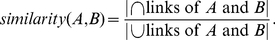
(2)The similarity is the overlap between the groups' connections and it estimates network proximity of the groups. The general pattern is that links with mentions more likely occur between close groups and retweets occur between groups with medium similarity (Figure 4D). Mentions as personal messages are typically exchanged between users with similar environments, what is predicted by the strength of weak ties theory. Links with retweets are related to information transfer and the similarity of the groups between which they take place should be small according to the Granovetter's theory. The results show that the most likely to attract retweets are the links connecting groups that are neither too close nor too far. This can be explained with Aral's theory about the trade-off between diversity and bandwidth: if the two groups are too close there is no enough diversity in the information, while if the groups are too far the communication is poor. These trends are not dependant on the size of the considered groups (see Figs. S6, S7, S8, S9, S10, S11, S12, S13, S14 and Table S1 in the Supplementary Information).

### 2.5 Intermediary links

**Figure 5:**
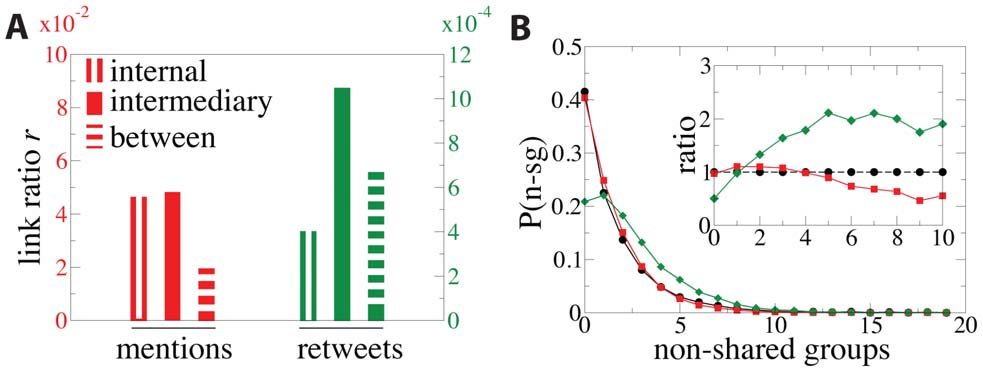
Intermediary links. (A) Ratio 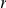 between the number of links with mentions or retweets and number of follower links. (B) Distribution of the links in the follower network (black curve), those with mentions (red curve) and retweets (green curve) as a function of the number of non-shared groups of the users connected by the link. Inset, ratios between these distributions and the follower network.

The communication between groups can take place in two ways: the information can propagate by means of links between groups or by passing through an intermediary user belonging to more than one group. We have defined as intermediary the links connecting a pair of users sharing a common group and with at least one of the users belonging also to a different group (see Fig. 1B). These users and their links have a high potential to pass information from one group to another in an efficient way [13]. Several previous works pointed out to the existence of special users in Twitter regarding the communication in the network [28], [48]. In order to estimate the efficiency of the different types of links as attractors of mentions and retweets, we measure a ratio 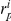 for links in position 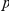 and for interaction 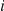 defined as
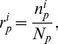
(3)where, as before, 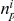 is the number of links with the interaction 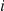 in position 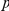 and 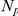 is the total number of links in that position. The bar plot with the values of 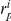 is displayed in Figure 5A. The efficiency of the different type of links can thus be compared for the attraction of mentions (red bars) and retweets (green bars). Links internal to the groups attract more mentions and less retweets than links between groups in agreement with the predictions of the strength of weak ties theory. Intermediary links attract mentions as likely as internal links: the fraction of intermediary links with mentions is very close to the fraction of internal links with mentions. This is expected because intermediary links are also internal to the groups. However, the aspect that differentiates more intermediary links from other type of links is the way that they attract retweets. Intermediary links bear retweets with a higher likelihood than either internal or between-groups connections (see Figure 5A and Figs. S1, S2, S3, and S4 in the Supplementary Information). This fact can be interpreted within the framework of the tradeoff between diversity and bandwidth [19]: strong ties are expected to be internal to the groups and to have high bandwidth, while ties connecting diverse environments or groups are more likely to propagate new information. High bandwidth links in our case correspond to those with multiple mentions, while links providing large diversity are the ones between groups. Intermediary links exhibit these two features: they are internal to the groups and statistically bear more mentions, and introduce diversity through the intermediary user membership in several groups. Although some theoretical works [12], [19] suggest that ties with high bandwidth and high diversity should be scarce, we find that intermediary links are as abundant as internal links (see Fig. 2C). Moreover, in line with the theories [12], [16], [19], higher diversity increases the chances for a link to bear retweets as can be seen in Figure 5B, which implies a more efficient information flow. In the inset of the Figure it is shown that the number of non-shared groups assigned to the users connected by the link positively correlates with a higher than expected number of retweets.

## Discussion

In summary, we have found groups of users analyzing the follower network of Twitter with clustering techniques. The activity in the network in terms of the messages called mentions and retweets clearly correlates with the landscape that the presence of the groups introduces in the network. Mentions, which are supposed to be more personal messages, tend to concentrate inside the groups or on links connecting close groups. This effect is stronger the larger the number of mentions exchanged and if they are reciprocated. Retweets, which are associated to information propagation events, appear with higher probability in links between groups, especially those that connect groups that do not show a high overlap, and more importantly on links connected to users who intermediate between groups. These intermediary users belong to multiple groups and play an important role in the spreading of information. They acquire information in one group and launch retweets targeting the other groups of which they are members. At the same time, the access to new information can transform them into attractive targets to be retweeted by their followers. The relevance of certain users for the spread of information in online social media has been discussed in previous works. Our method provides a way to identify these special users as brokers of information between different groups using as only input the follower network.

From the sociological point of view, the way that the activity localizes with respect to the groups allow us to establish a parallelism with the organization of offline social networks.In particular, we have shown that the theory of the strength of weak ties proposed by Granovetter to characterize offline social network applies also to an online network. Furthermore, some of our results can be explained within the framework of Burt's brokerage and closure and Aral's diversity-bandwidth tradeoff theories. The specific properties of Twitter offers an opportunity to study directly the importance of the links for personal communications or for information diffusion. According to these theories, the strong social ties tend to appear at the interior of the groups or between close groups as happens for the links with mentions in Twitter. In addition, the socially weak ties are expected to be more common connecting different groups and to be important for the propagation of information in the network. This is similar to what we observe for the links with retweets that concentrate with high probability in links between dissimilar groups or in intermediary links. Besides the roles assigned by these two theories to the links, we have found that intermediary users and links are also an important component to take into account for understanding information propagation. These links tend to be characterized by high bandwidth and diversity in the context of Aral's study, and exhibit high information diffusion efficiency. Based on all these findings, despite the myth of one million friends and the doubts on the social validity of online links, the simplest connections of the online network bear valuable information on where higher quality interactions take place.

## Materials and Methods

### 4.1 Ethics Statement

The data analyzed are publicly available as they come from a public online social site (Twitter). Furthermore any private information has been removed from the database before the analysis, which has been performed using anonymized data.

### 4.2 Description of the dataset

The data analyzed in this paper was collected in a two step process: the fist stage corresponds to the collection of the *follower network* (followers and followees), while the second consists in the retrieval of the user activity from the stream of Twitter (plain tweets, mentions and retweets). In the first stage, the directed unweighted network is obtained from the information on the followers and followees of each user. The data was collected using a breadth-first search technique: Starting from several seeds, followers and followees of the seeds were retrieved. Then the same procedure was repeated for the newly discovered users obtaining a so-called snowball sampling of the follower network. The procedure is stopped after several steps when the number of newly discovered users in 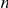-th breadth is small compared with the total number of users already discovered in the 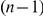-th step. The process was run in November 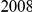, gathering information for a total of 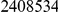 users. Due to the internal exploration of the network, one can anticipate that this method tends to detect the users with the highest in or out degree that belong to the largest connected cluster of the network.

The second stage consists in searching for all the tweets of the users found in the follower network for a period of time from November 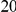 to December 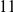. The activity dataset was constructed from these gathered tweets. The tweets containing usernames with a ‘@username’ functional syntax were used for the mentions. Tweets that were reposted from other users, and which also hold a special format of the form ‘RT @username’, were used to build our retweet dataset. In some cases for mentions and retweets multiple users can be specified. Then we count only the first user for the purpose of our analysis. It is also worthy to note that mentions (replies) and retweets are now implemented into Twitter system [23]. The subset of retweets has been removed from a set of mentions to avoid overlap. In total, we obtained 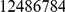 tweets from 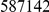 users in the network, what stands for 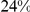 of all users from the follower network. The rest of users either did not posted any tweet in their profile during the period of data collection (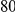
–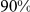 of cases), had a protected profile (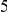
–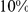 of cases) or removed their profiles (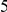
–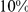 of cases). Out of these tweets 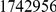 where mentions and 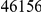 where retweets. For the purpose of the analysis we have filtered out mentions and retweets which happened without underlying follower relation, in order to avoid inclusion of messages sent to not-known users and also to be able to perform comparisons with our baseline model consisting of the follower network. The resulting set of links with different interactions is summarized in Table 1. Note, that links with mentions/retweets can have multiple mentions/retweets happening over them.

**Table 1:** Overall characteristics of the follower network and of the interactions taking place on it.

Property	Follower	Links with	Links with
	links	mentions	retweets
Users	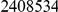	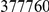	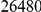
Links	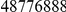	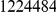	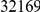
Reciprocity	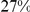	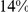	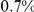

The dataset is a good representation of what Twitter was at the end of 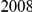 both in the social network and in the activity of the users. According to Ref. [49], Twitter at the time of the data collection had less than 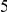 million registered users. Therefore we estimate that our dataset contains information about more than 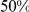 of the most active users from that time. Other aspects of this dataset related to system scalability and trace generation were studied in Refs. [30], [32], [33].

### 4.3 The OSLOM clustering method

OSLOM is a method based on a topological approach to detect statistically significant clusters [35], [36]. A null model that consists of graphs obtained by reshuffling the connections of the given network is considered. As a next step the probability of finding each group in the ensemble formed by these random graphs is estimated. During this procedure, it is assumed that an optimized clustering technique has been applied to the random graphs and therefore it is necessary to use techniques from the statistics of extremes and from order statistics to evaluate properly the probability of each group. Oslom incorporates a local search method for the exploration of the network with the aim of finding clusters that improve the estimated probability, that is to find groups that have lower probability of existence in random graphs. OSLOM provides a set of clusters at the lowest hierarchical level and a list of nodes belonging to several groups and those not belonging to any group. The method has been tested in different benchmark networks containing planted groups, nodes belonging to several groups and nodes added to the network with random connections. Its high level of proficiency to recover the planted groups has been proved even when nodes with random connections are introduced in a graph with bona fide group structure. In those cases, OSLOM detects these nodes as no-group nodes [35].

## Supporting Information

Figure S1 **Percentage of links of different types, e.g. follower links (black bars), links with mentions (red bars) or retweets (green bars), staying in particular topological localizations in respect to detected groups.** The locations of links with respect to the groups correspond to those shown in Figure 1D of the main paper. This gure corresponds to Figure 2C in the main paper.
(PDF)

Figure S2 **Averaged group-group similarity for groups paired by follower links as a function of the groups sizes.**
(PDF)

Figure S3 Ratio between the average group similarity for the between-group links with mentions (A) or retweets (B) and the follower network as function of the size of the group of origin and destination.
(PDF)

Figure S4(A) Fraction of links in the follower network, of links with mentions and links with retweets for bridges as a function of the size of the group. This figure is equivalent to the Figure 2A of the main paper but for bridges instead of pure internal links. (B) Fraction of links with mention activity of different intensity. The dashed curves are the total for the follower network (black) and for the links with mentions (red). While the other curves correspond (from bottom to top) to fractions of links with: one non-reciprocated mention (diamonds), 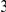 mentions (circles), 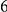 mentions (triangle up) and more than 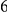 mentions (triangle down).(PDF)

Figure S5
**Normalized mutual information as a function of the ratio between the number of links between groups and internal links to the groups in a benchmark.** The benchmark is composed of 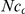 cliques (fully connected subgraphs) o0f size Sc each.(PDF)

Figure S6Internal activity for different clustering algorithms from left up corner to the right: Oslom, Infomap, Moses, Louvain, Real-time community detection, and Radatools. Fraction of links of different types internal to the groups as a function of the group size in number of users. The black curve is for the follower network, which acts as baseline for the links with any mentions (red curve with closed square symbols) and for links with specific number of mentions (red curves with open triangle symbols rotated 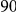 degrees counterclockwise starting from straight up triangle: one mention non-reciprocated, 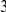 mentions, 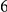 mentions, and more than 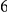 mentions reciprocated).(PNG)

Figure S7Internal activity for different clustering algorithms run for the snowball sample of the network (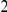 neighbors away from a random seed), from left up corner to the right: Oslom, Infomap, Moses, Louvain, Real-time community detection, and Radatools.(PNG)

Figure S8Internal activity for different clustering algorithms run for the snowball sample of the network (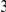 neighbors away from a random seed), from left up corner to the right: Oslom, Infomap, Moses, Louvain, Real-time community detection, and Radatools.(PNG)

Figure S9Internal activity for different clustering algorithms run for the subgraph of randomly chosen 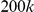 nodes, from left up corner to the right: Oslom, Infomap, Moses, Louvain, Real-time community detection, and Radatools.(PNG)

Figure S10 Internal activity for different clustering algorithms run for the network with removed hubs, from left up corner to the right: Oslom, Infomap, Moses, Louvain, Real-time community detection, and Radatools.
(PNG)

Figure S11Internal activity for different clustering algorithms run for the subgraph build from 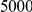 randomly selected groups found by Oslom, from left up corner to the right: Oslom, Infomap, Moses, Louvain, Real-time community detection, and Radatools.(PNG)

Figure S12 **Activity on between-groups links when the groups are detected by Infomap in the sample without hubs.** The panel reproduces the structure of Figure 3 of the main paper and of Figure S3. (A) Fraction of links in the follower networks as a function of the size of the group of origin and destination. (B) and (C) Fraction of links of different types: follower relations (black circles), links with mentions (red squares) or with retweets (green diamonds), as a function of the size of the group of origin or destination, respectively. (D) Frequency of links of the different types as a function of the group-group similarity. Ratio between the average group similarity for the links between groups with mentions (E) or retweets (F) and the follower network as function of the size of the group of origin and destination.
(PDF)

Figure S13 Bridges between groups detected by Moses for the network sample without hubs. (A) Distribution of the links in the follower network (black curve), those with mentions (red curve) and retweets (green curve) as a function of the number of not-shared groups of the users at the extreme of the link. (B) Ratio between these distributions taking the follower network as baseline. (C) Distribution of the number of groups to which each user is assigned.
(PNG)

Figure S14 **Jaccard similarity of users followers.** Users similarity frequency for pairs of users connected by a follower link (black circles), by a link with a mention (red squares) and a link with retweet (green diamonds). Inset: ratio between these frequencies taking the follower network as a baseline.
(PNG)

Table S1
**Summary of the results regarding internal connections when the groups are obtained with several clustering algorithms for different samples of the network.** We measure the trend of the mentions to concentrate in internal connections. Legend: 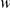 - weak signal, 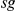 - signal only for small groups, typically smaller than 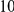 members, a hyphen is inserted if we have no results.(PDF)
