# Supplementary figures and images for: Social Features of Online Networks: The Strength of Intermediary Ties in Online Social Media
Source: PLoS One. 2012 Jan 11;7(1):e29358. doi: 10.1371/journal.pone.0029358 (PMC3256152; doi:10.1371/journal.pone.0029358)

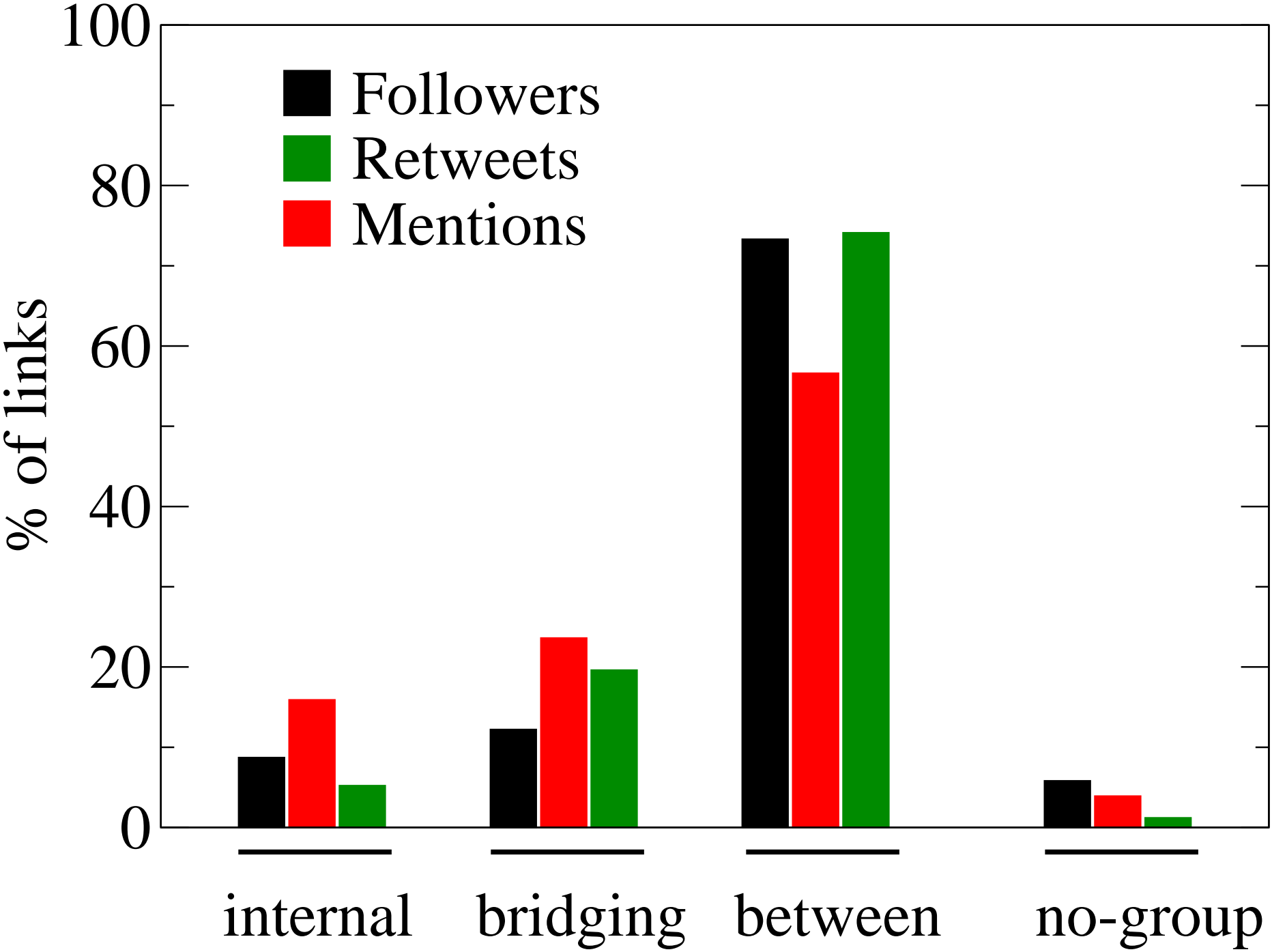

Supplement: Figure S1 — Percentage of links of different types, e.g. follower links (black bars), links with mentions (red bars) or retweets (green bars), staying in particular topological localizations in respect to detected groups. The locations of links with respect to the groups correspond to those shown in Figure 1D of the main paper. This gure corresponds to Figure 2C in the main paper. (PDF) [file pone.0029358.s001.pdf]

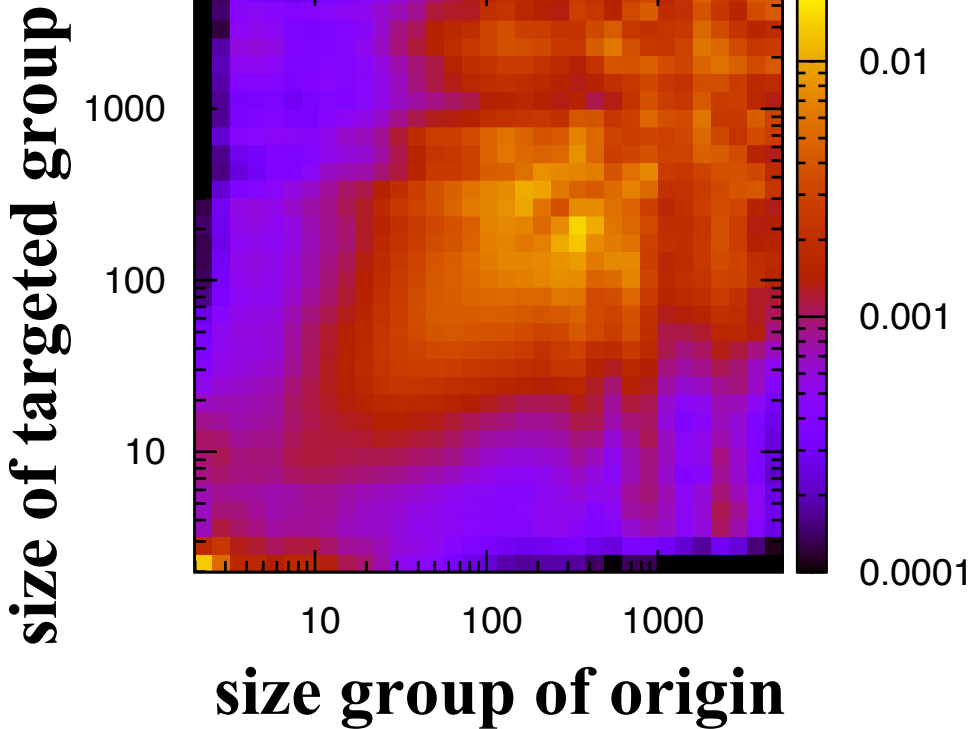

Supplement: Figure S2 — Averaged group-group similarity for groups paired by follower links as a function of the groups sizes. (PDF) [file pone.0029358.s002.pdf]

**A****Mentions/Followers**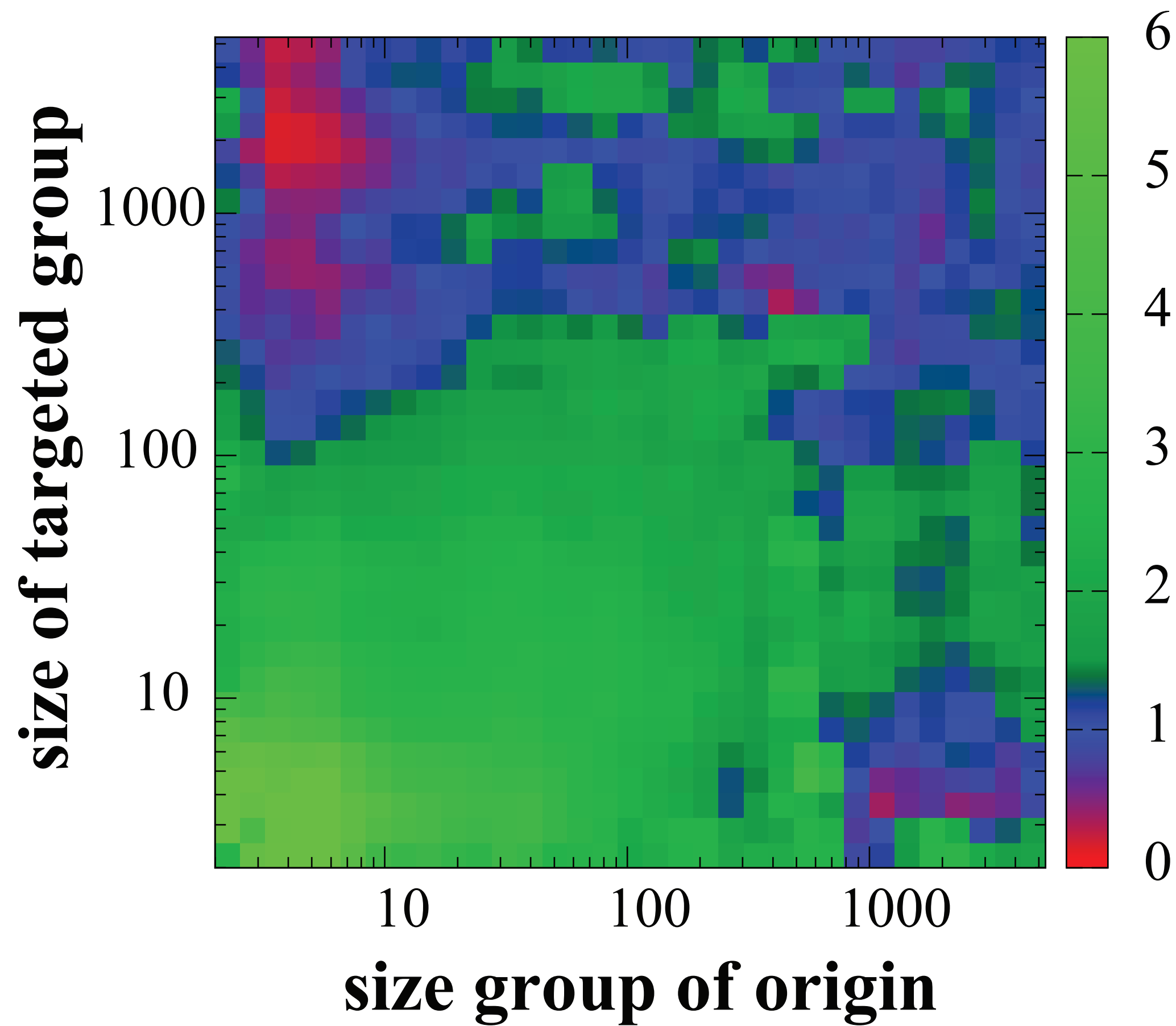**B****Retweets/Followers**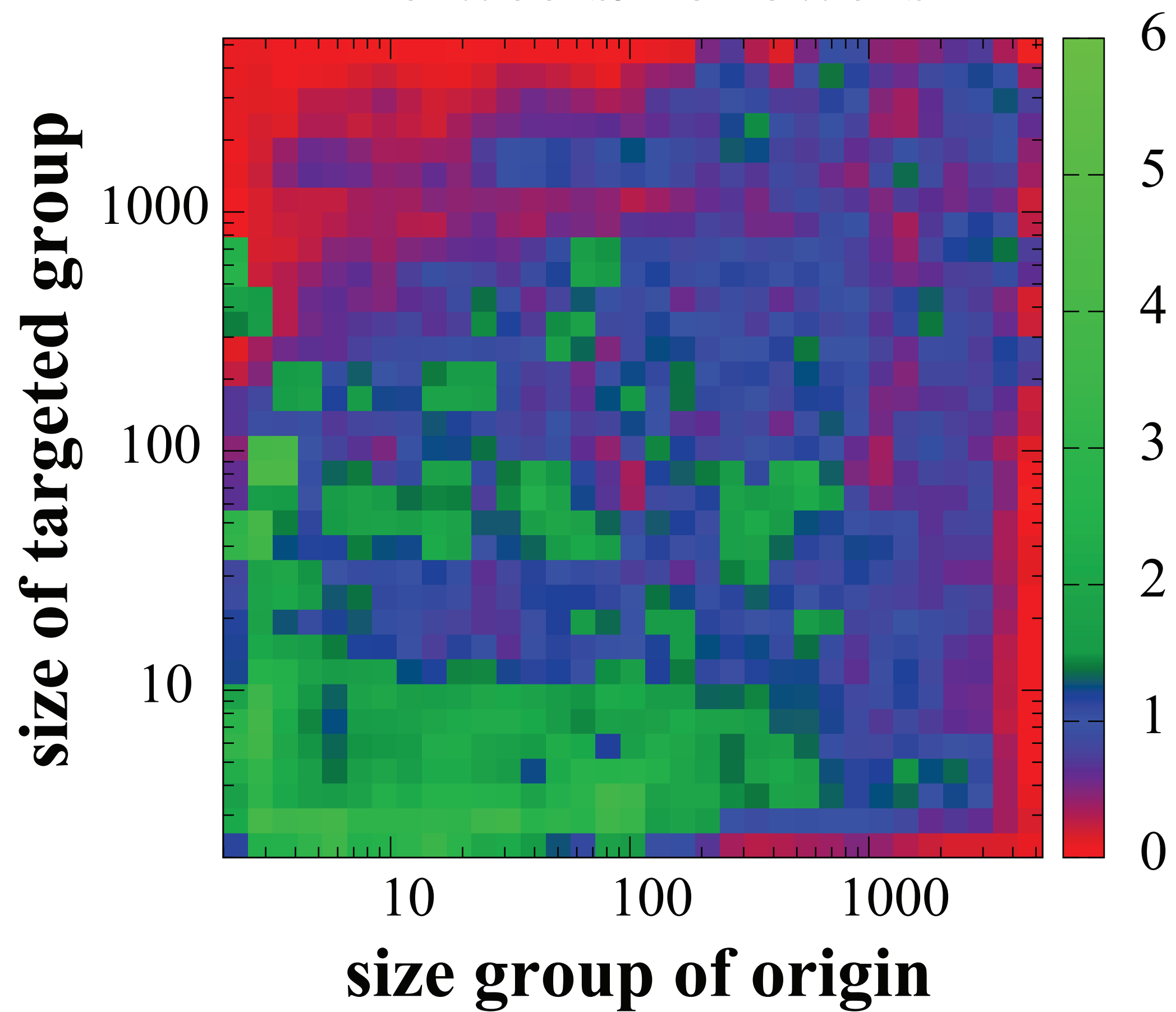

Supplement: Figure S3 — Ratio between the average group similarity for the between-group links with mentions (A) or retweets (B) and the follower network as function of the size of the group of origin and destination. (PDF) [file pone.0029358.s003.pdf]

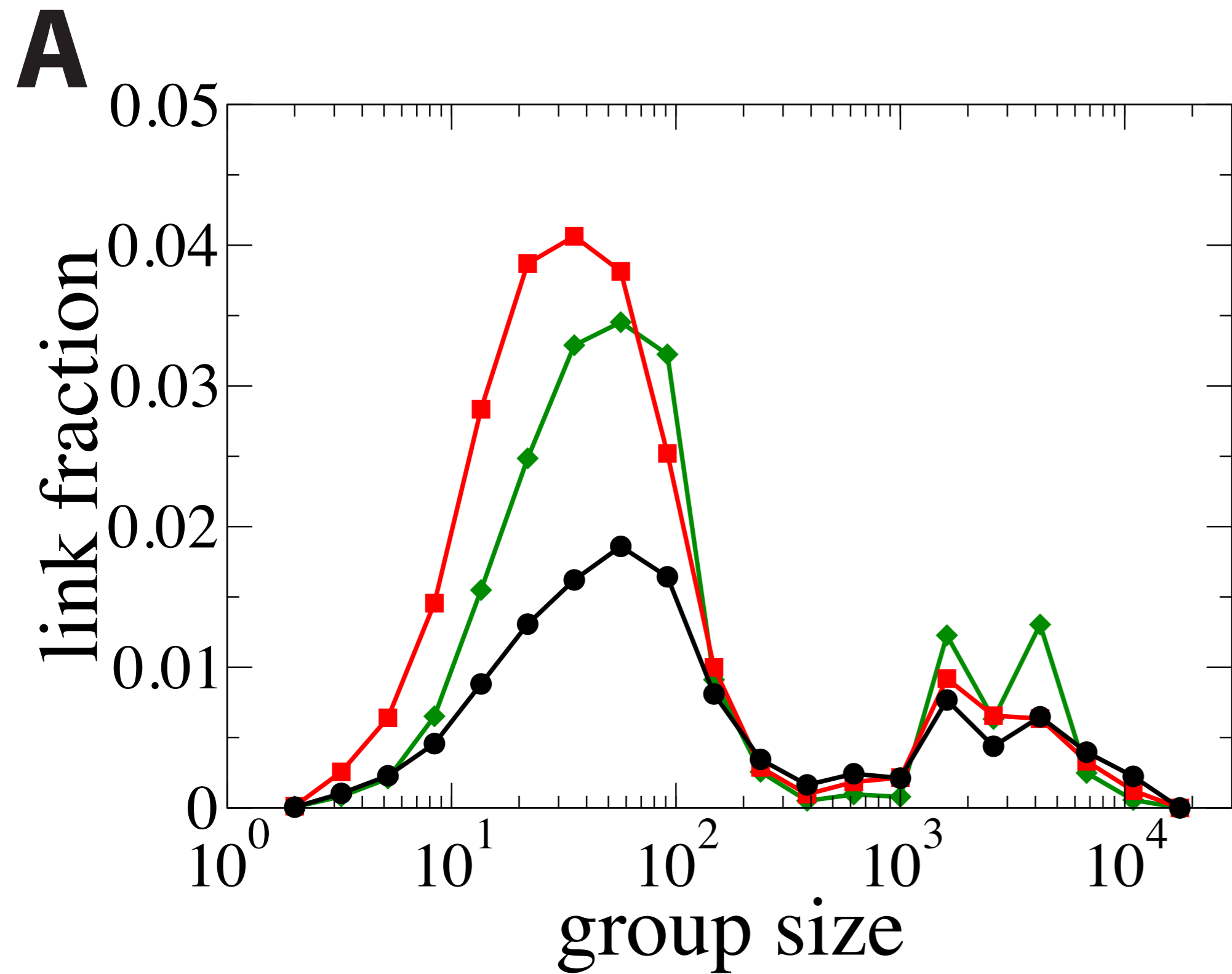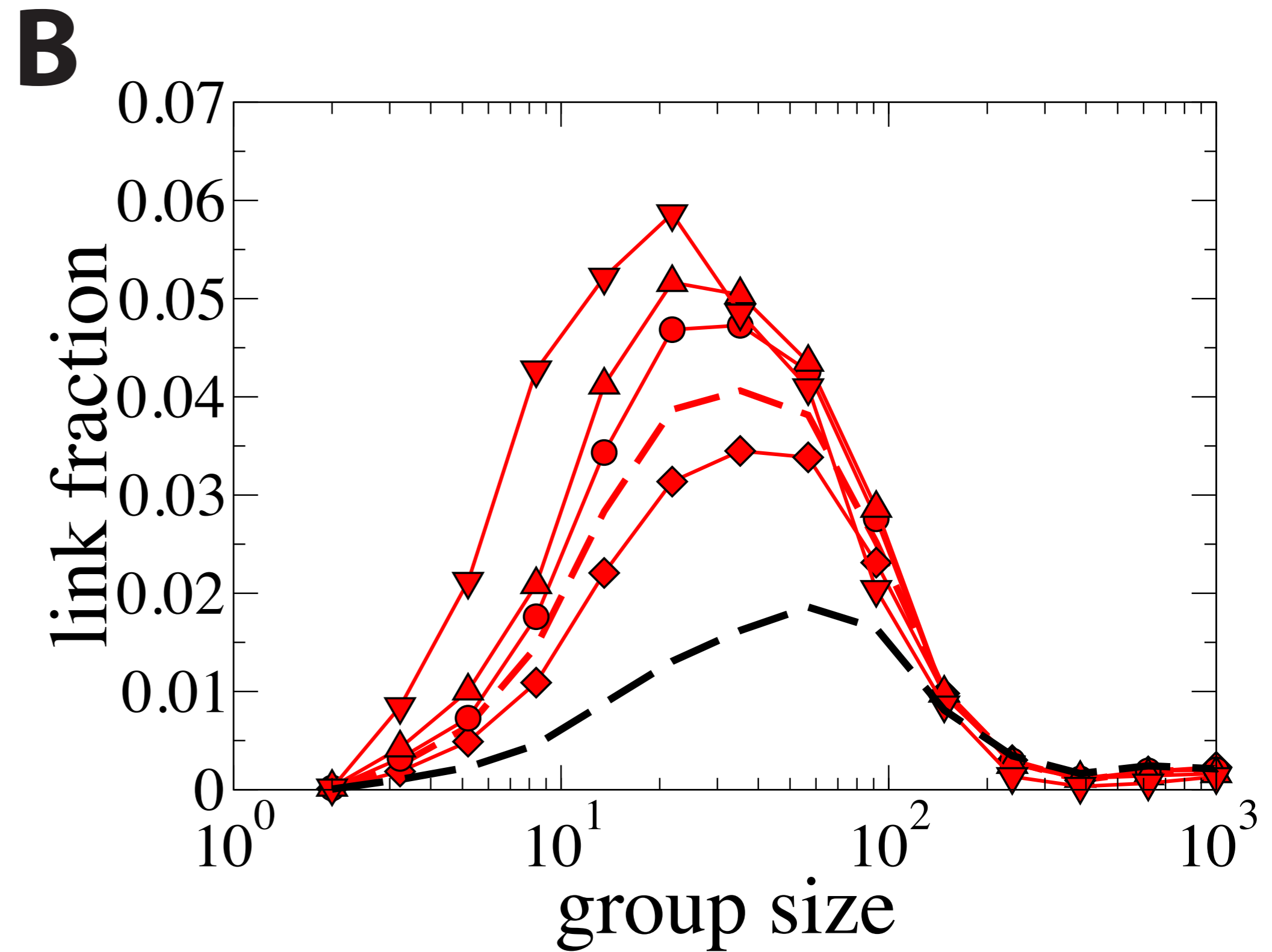

Supplement: Figure S4 — (A) Fraction of links in the follower network, of links with mentions and links with retweets for bridges as a function of the size of the group. This figure is equivalent to the Figure 2A of the main paper but for bridges instead of pure internal links. (B) Fraction of links with mention activity of different intensity. The dashed curves are the total for the follower network (black) and for the links with mentions (red). While the other curves correspond (from bottom to top) to fractions of links with: one non-reciprocated mention (diamonds), mentions (circles), mentions (triangle up) and more than mentions (triangle down). (PDF) [file pone.0029358.s004.pdf]

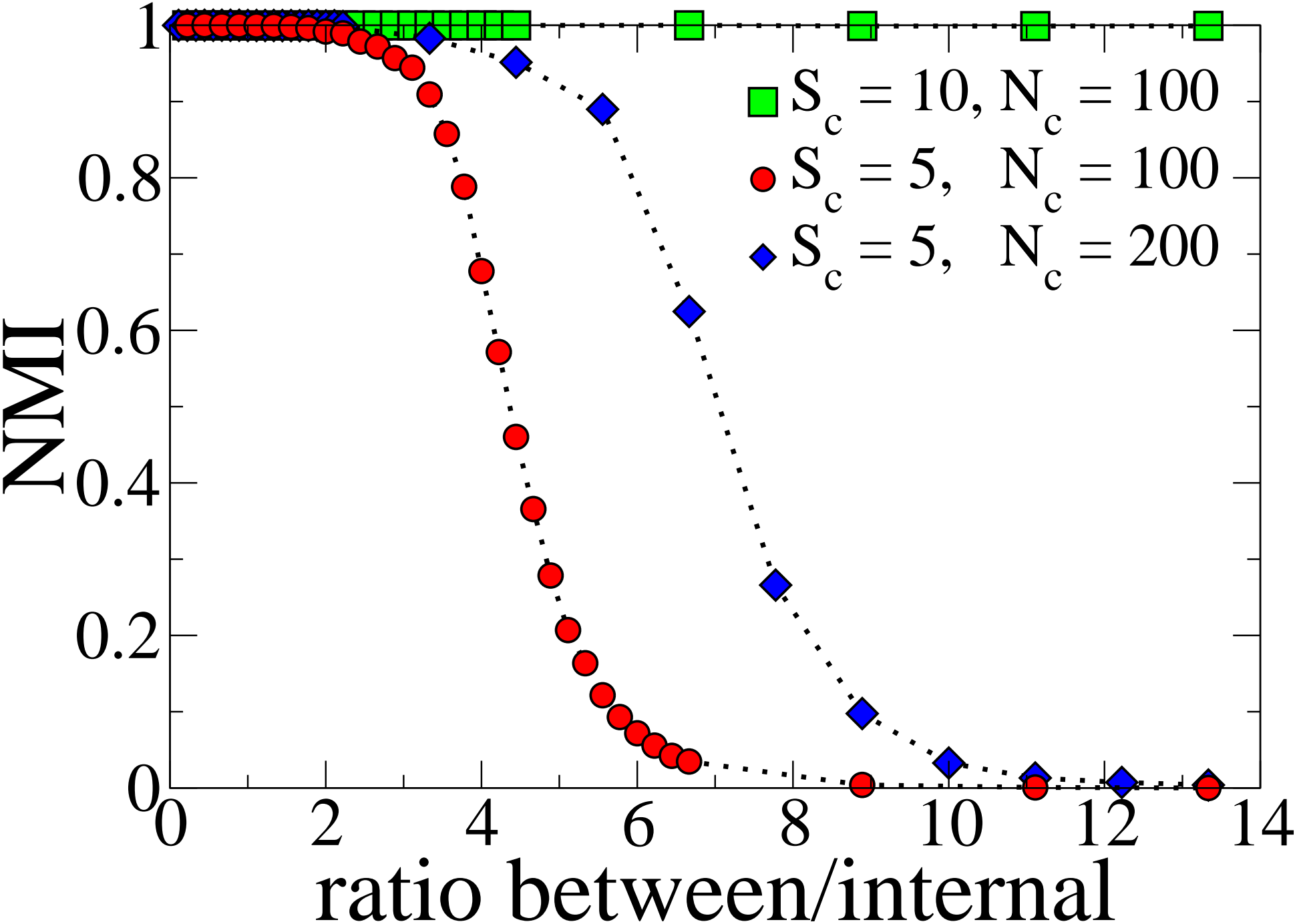

Supplement: Figure S5 — Normalized mutual information as a function of the ratio between the number of links between groups and internal links to the groups in a benchmark. The benchmark is composed of cliques (fully connected subgraphs) o0f size Sc each. (PDF) [file pone.0029358.s005.pdf]

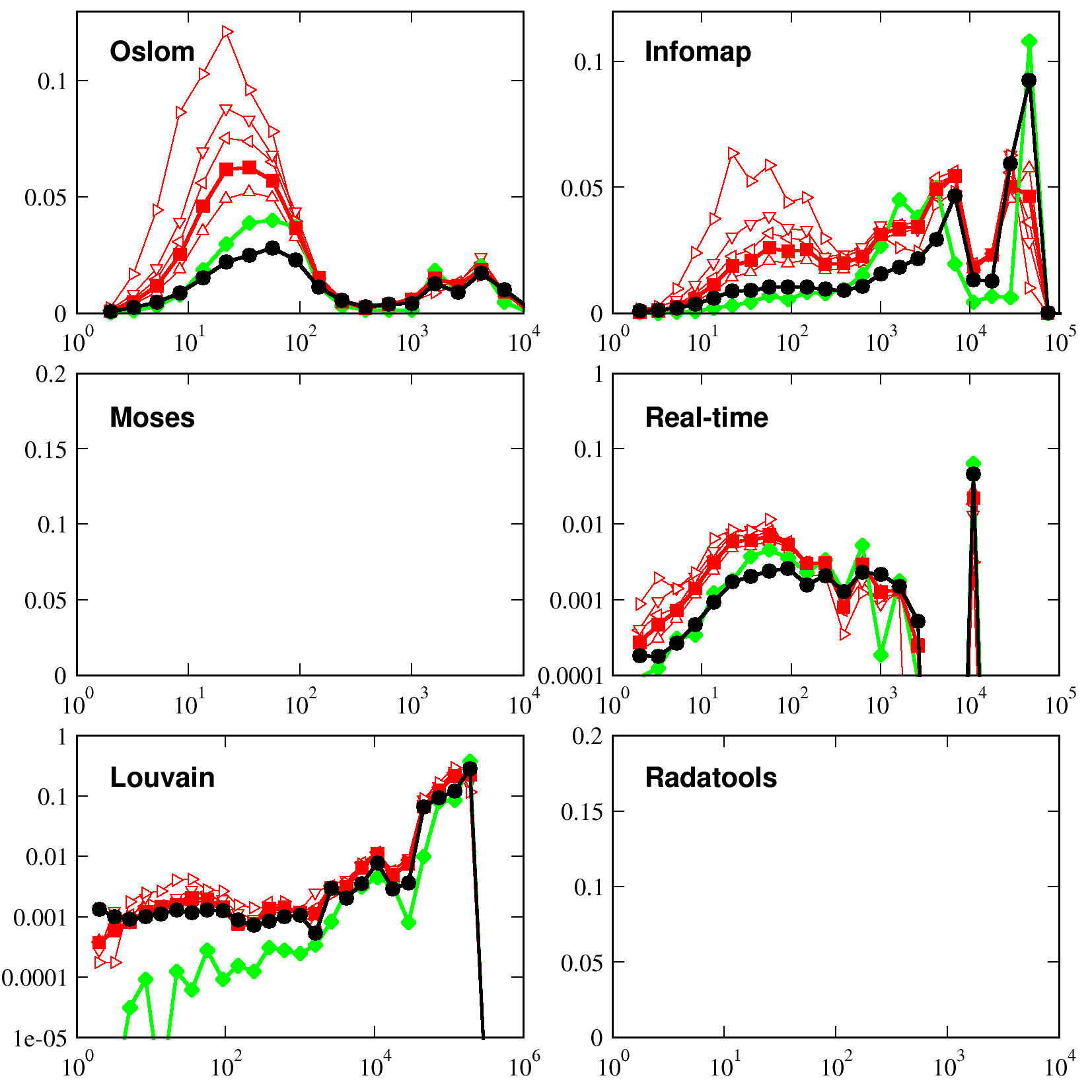

Supplement: Figure S6 — Internal activity for different clustering algorithms from left up corner to the right: Oslom, Infomap, Moses, Louvain, Real-time community detection, and Radatools. Fraction of links of different types internal to the groups as a function of the group size in number of users. The black curve is for the follower network, which acts as baseline for the links with any mentions (red curve with closed square symbols) and for links with specific number of mentions (red curves with open triangle symbols rotated degrees counterclockwise starting from straight up triangle: one mention non-reciprocated, mentions, mentions, and more than mentions reciprocated). (PNG) [file pone.0029358.s006.png]

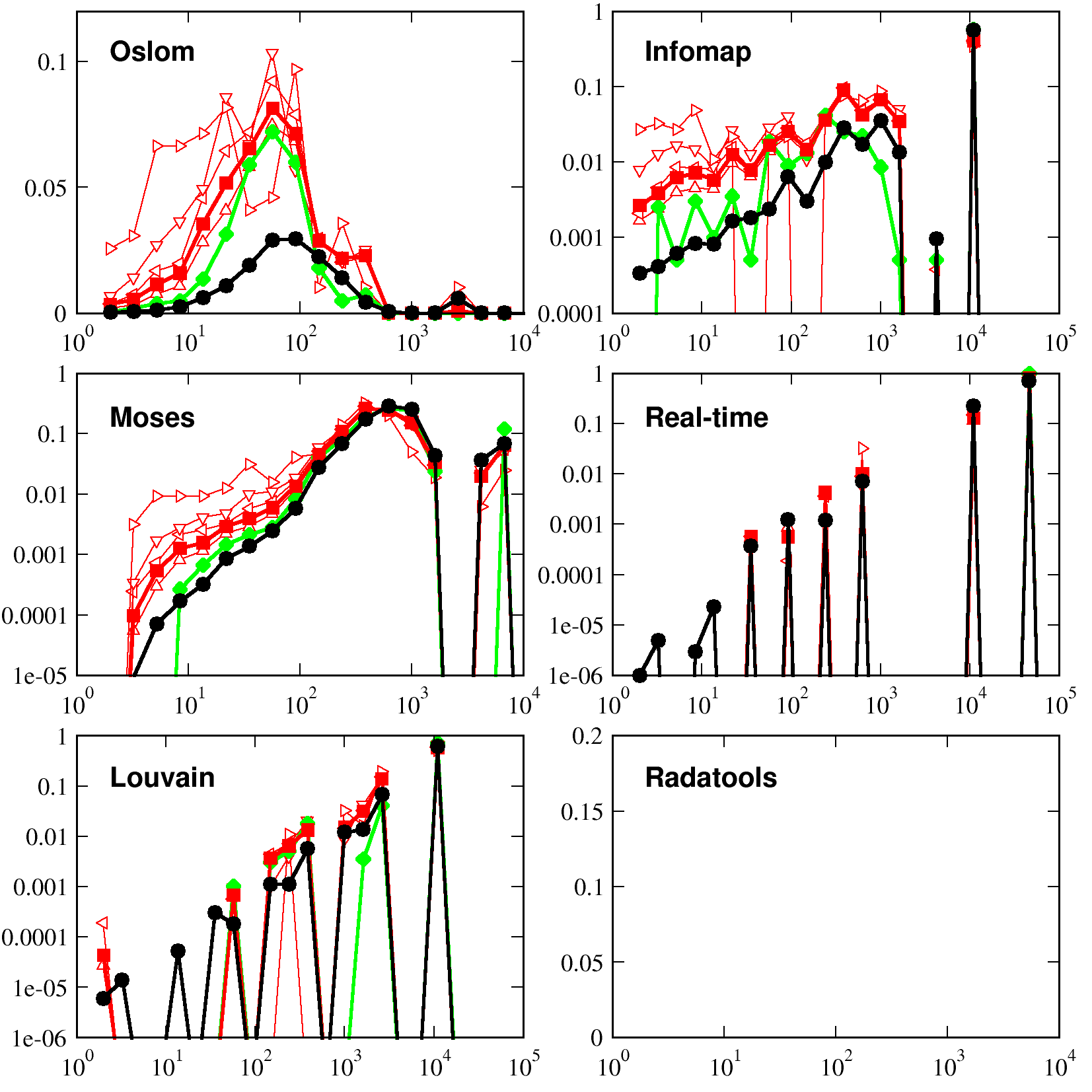

Supplement: Figure S7 — Internal activity for different clustering algorithms run for the snowball sample of the network ( neighbors away from a random seed), from left up corner to the right: Oslom, Infomap, Moses, Louvain, Real-time community detection, and Radatools. (PNG) [file pone.0029358.s007.png]

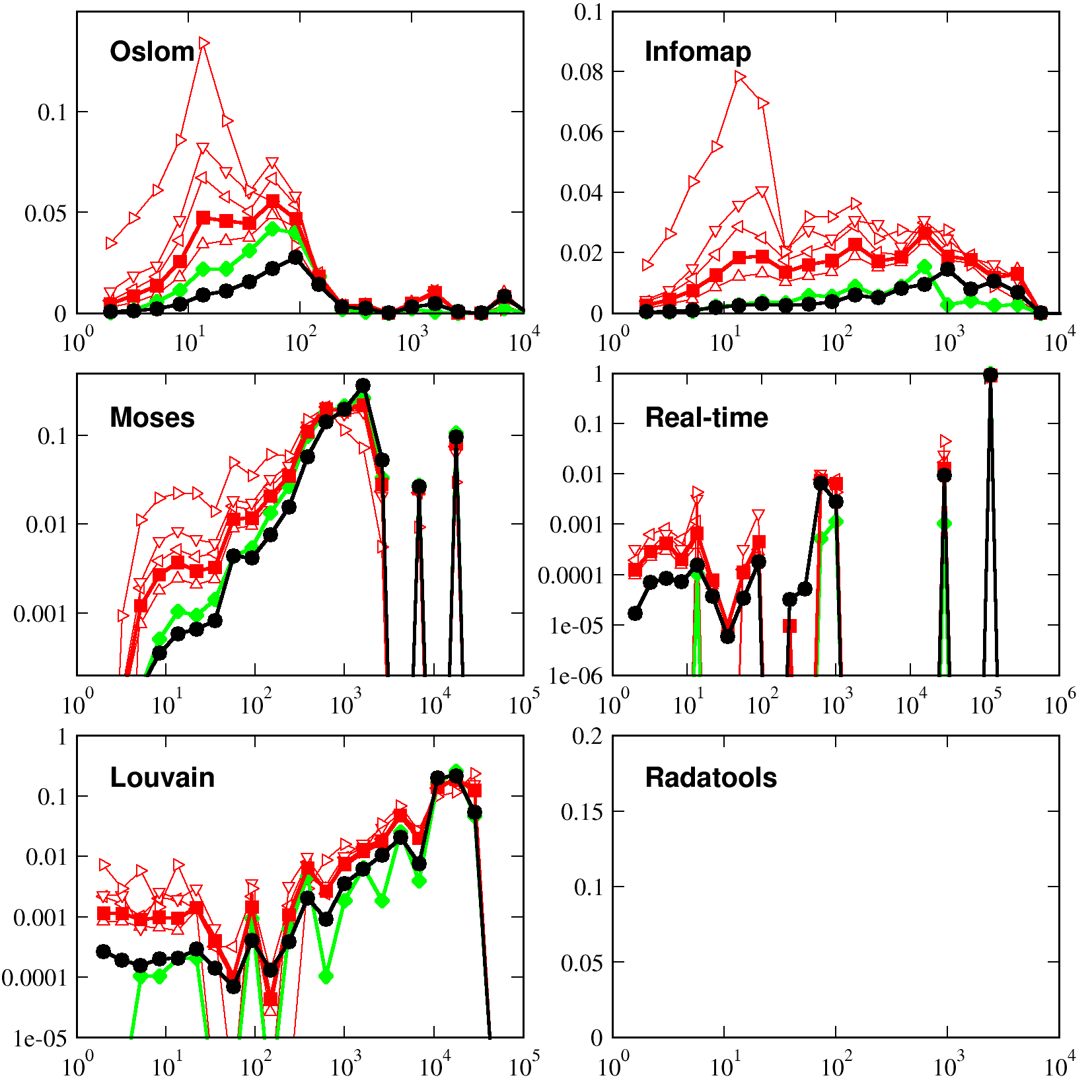

Supplement: Figure S8 — Internal activity for different clustering algorithms run for the snowball sample of the network ( neighbors away from a random seed), from left up corner to the right: Oslom, Infomap, Moses, Louvain, Real-time community detection, and Radatools. (PNG) [file pone.0029358.s008.png]

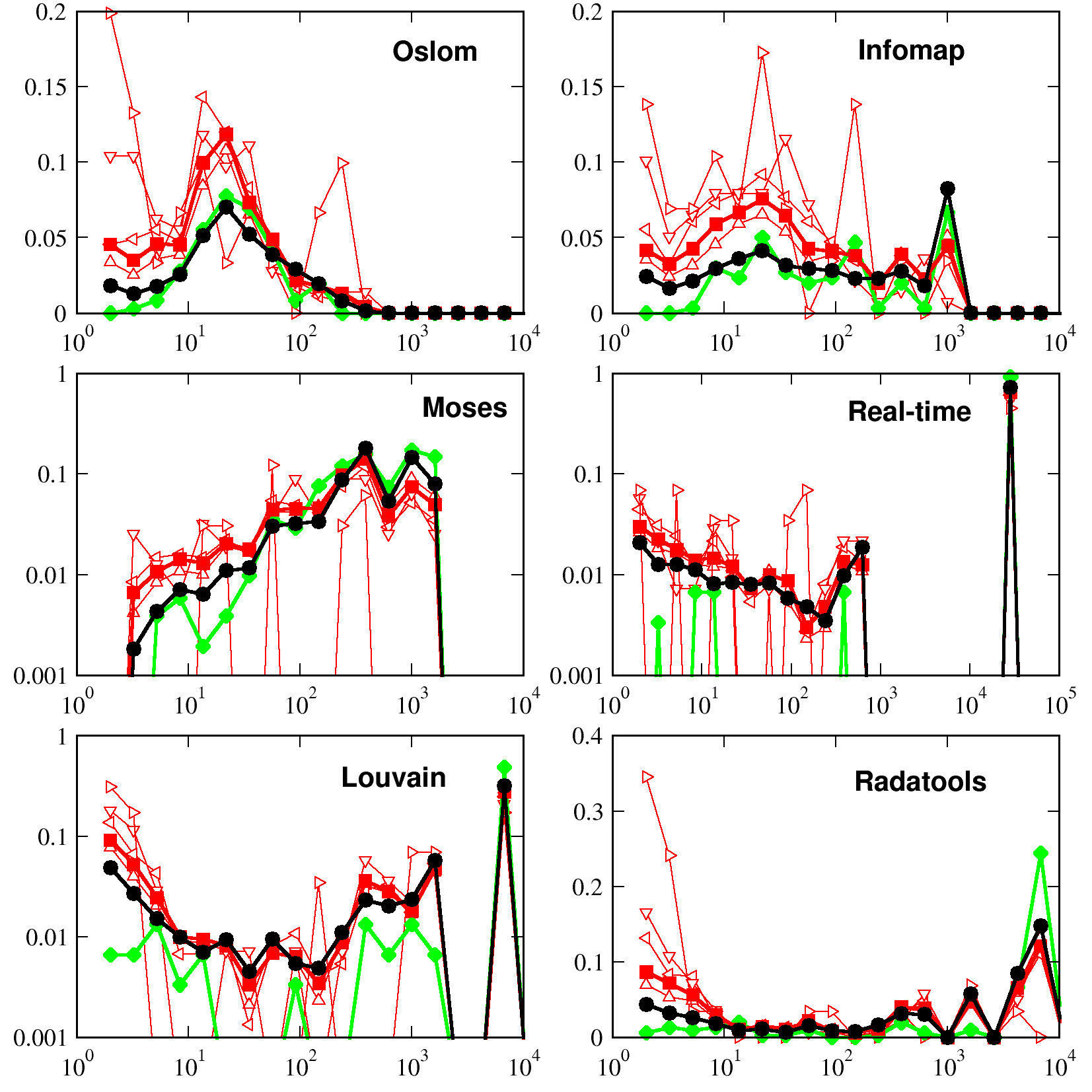

Supplement: Figure S9 — Internal activity for different clustering algorithms run for the subgraph of randomly chosen nodes, from left up corner to the right: Oslom, Infomap, Moses, Louvain, Real-time community detection, and Radatools. (PNG) [file pone.0029358.s009.png]

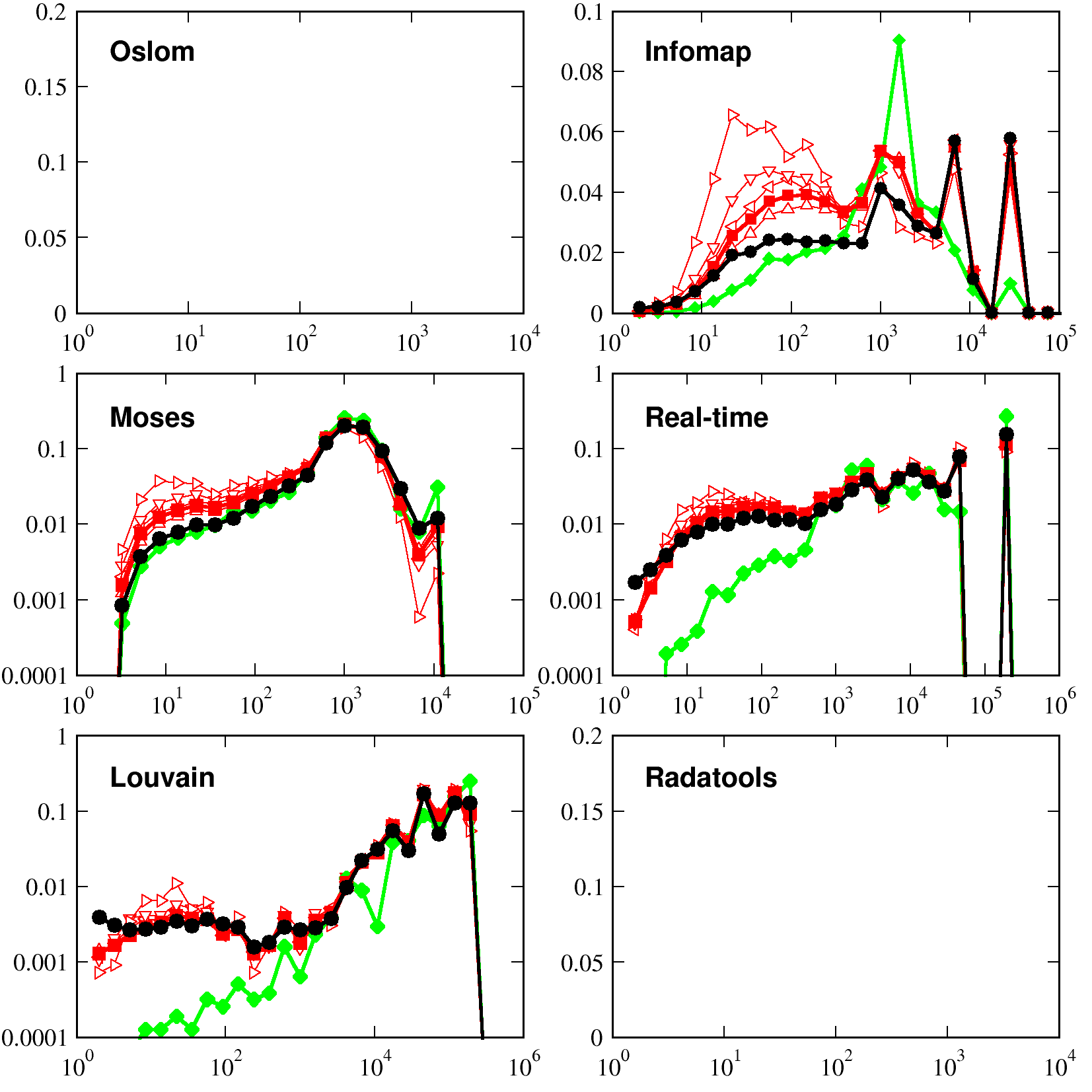

Supplement: Figure S10 — Internal activity for different clustering algorithms run for the network with removed hubs, from left up corner to the right: Oslom, Infomap, Moses, Louvain, Real-time community detection, and Radatools. (PNG) [file pone.0029358.s010.png]

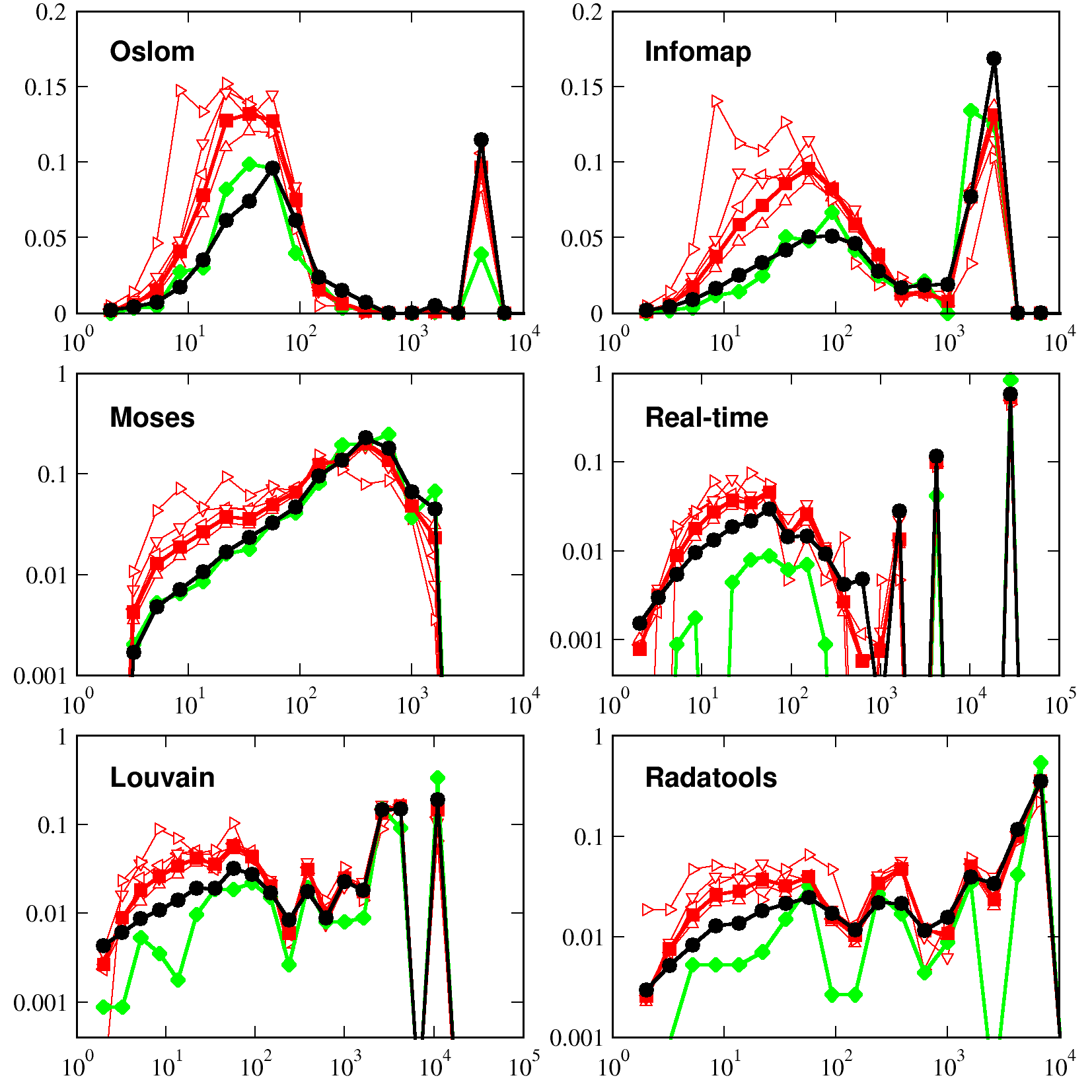

Supplement: Figure S11 — Internal activity for different clustering algorithms run for the subgraph build from randomly selected groups found by Oslom, from left up corner to the right: Oslom, Infomap, Moses, Louvain, Real-time community detection, and Radatools. (PNG) [file pone.0029358.s011.png]

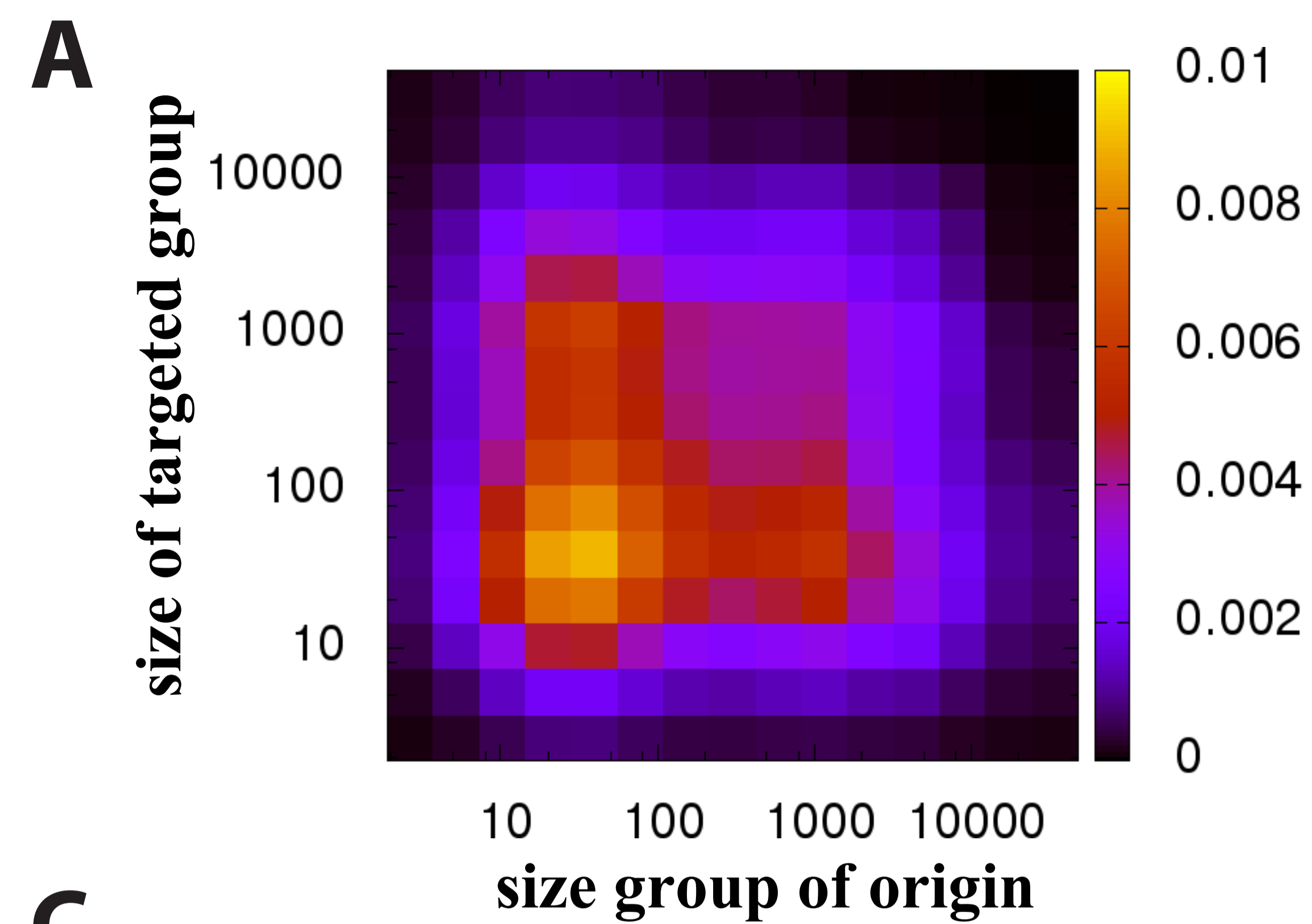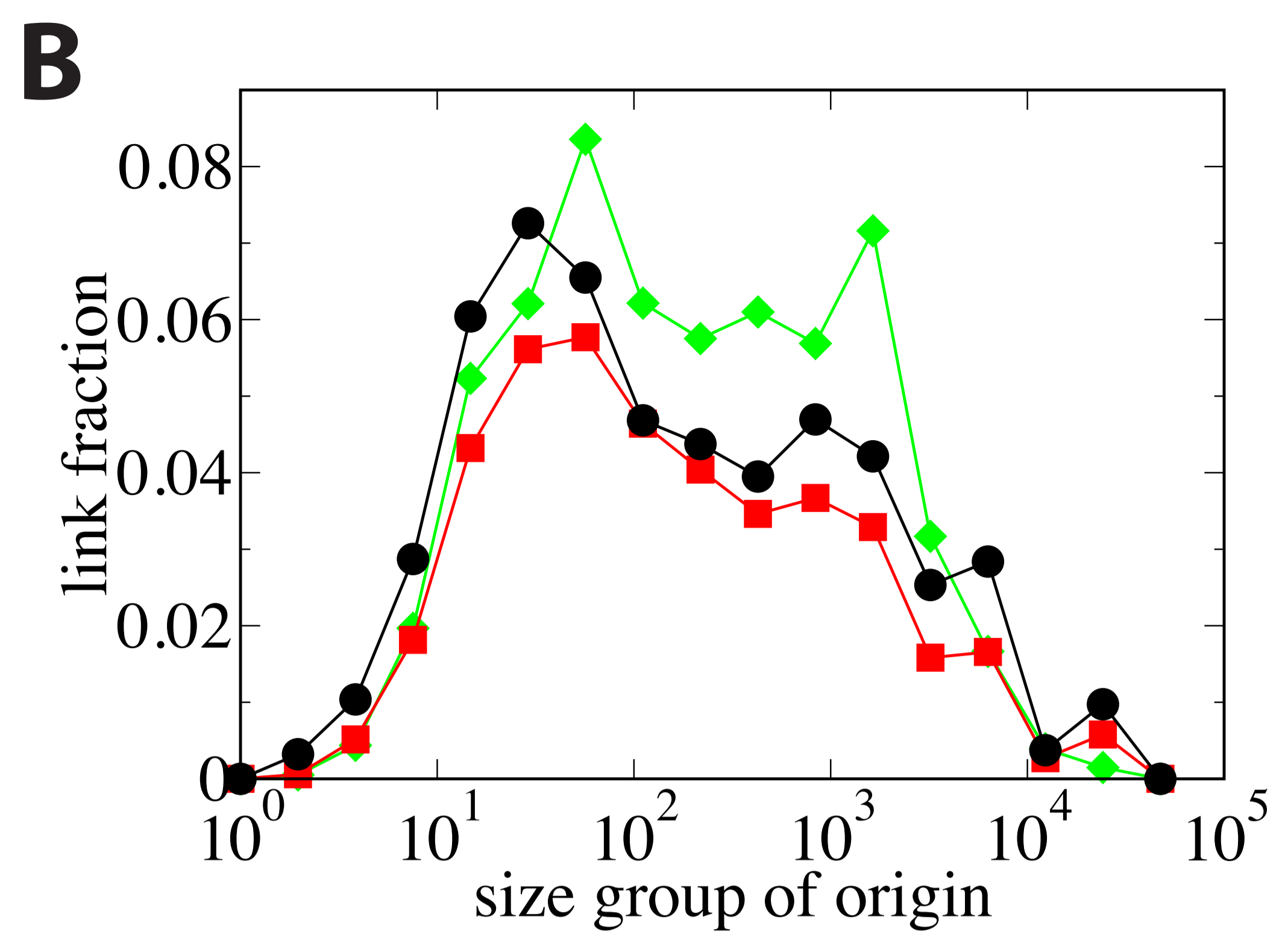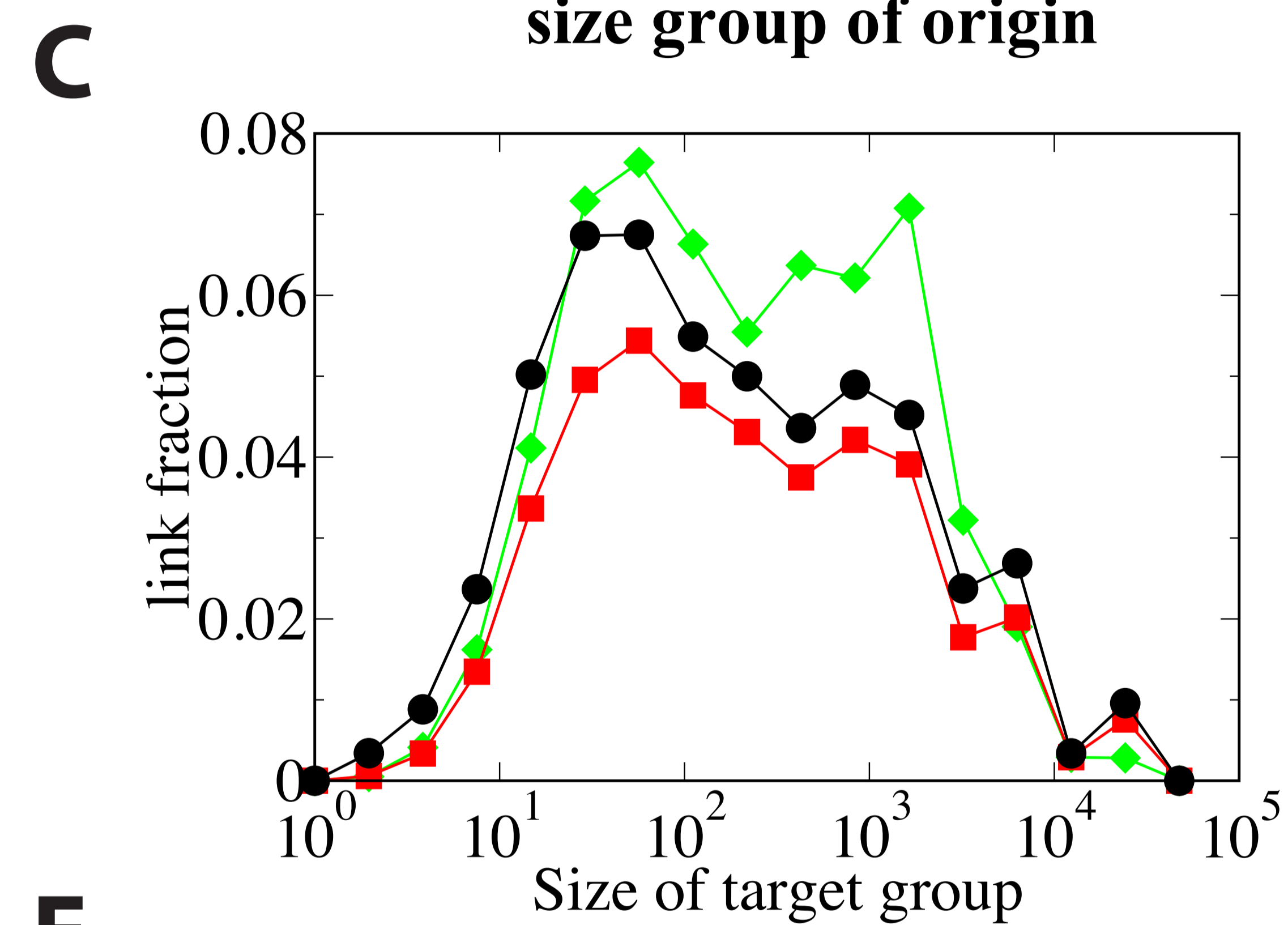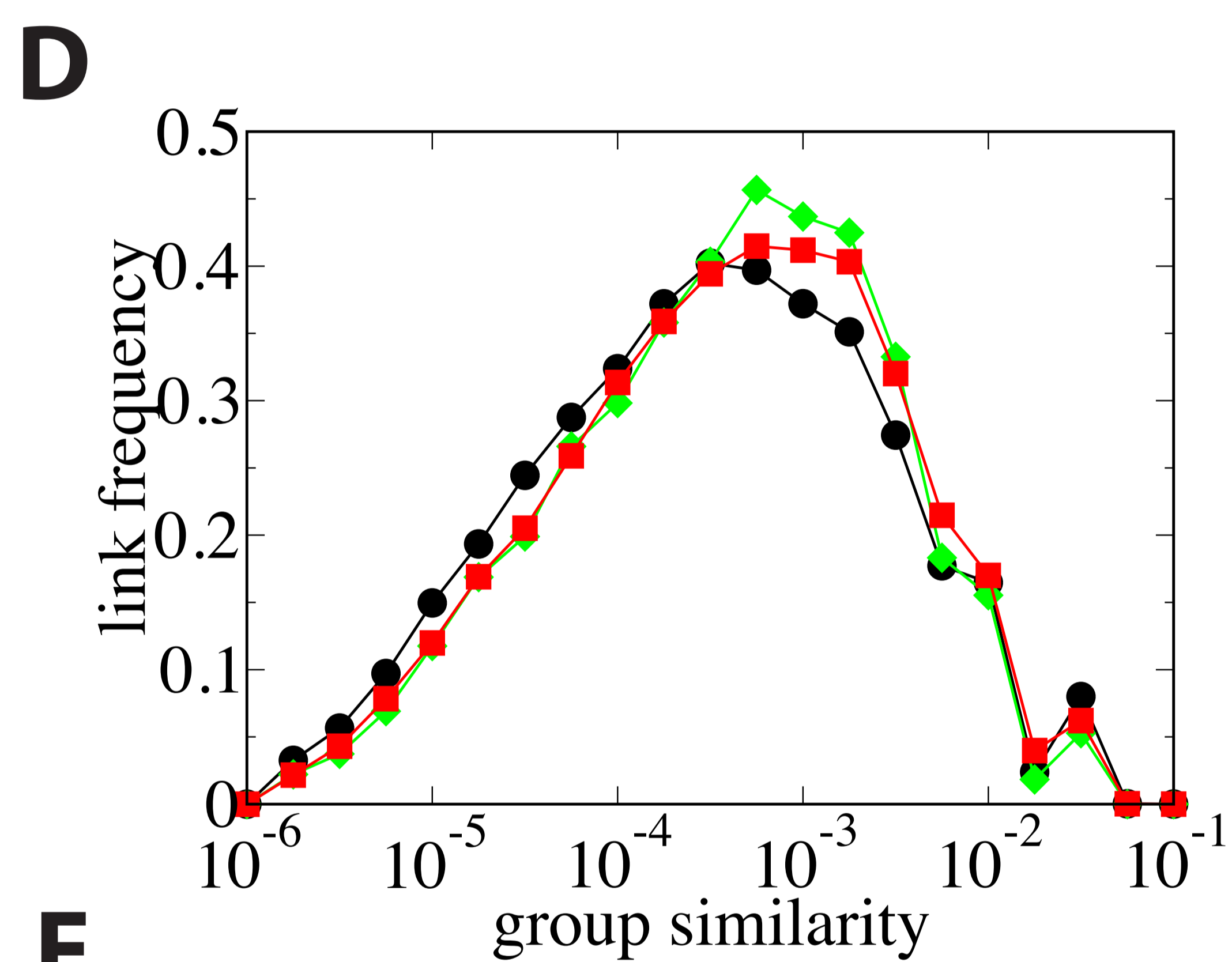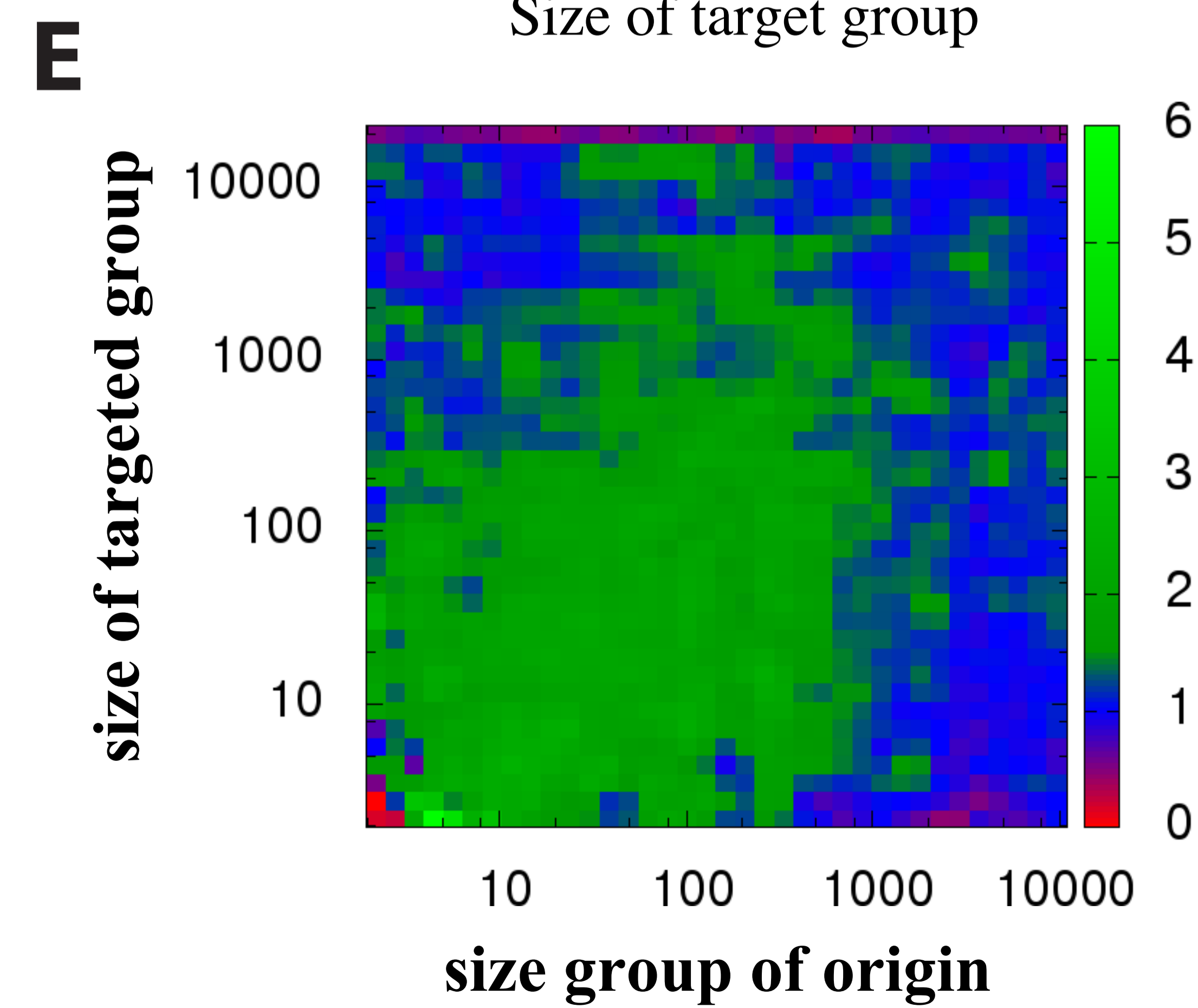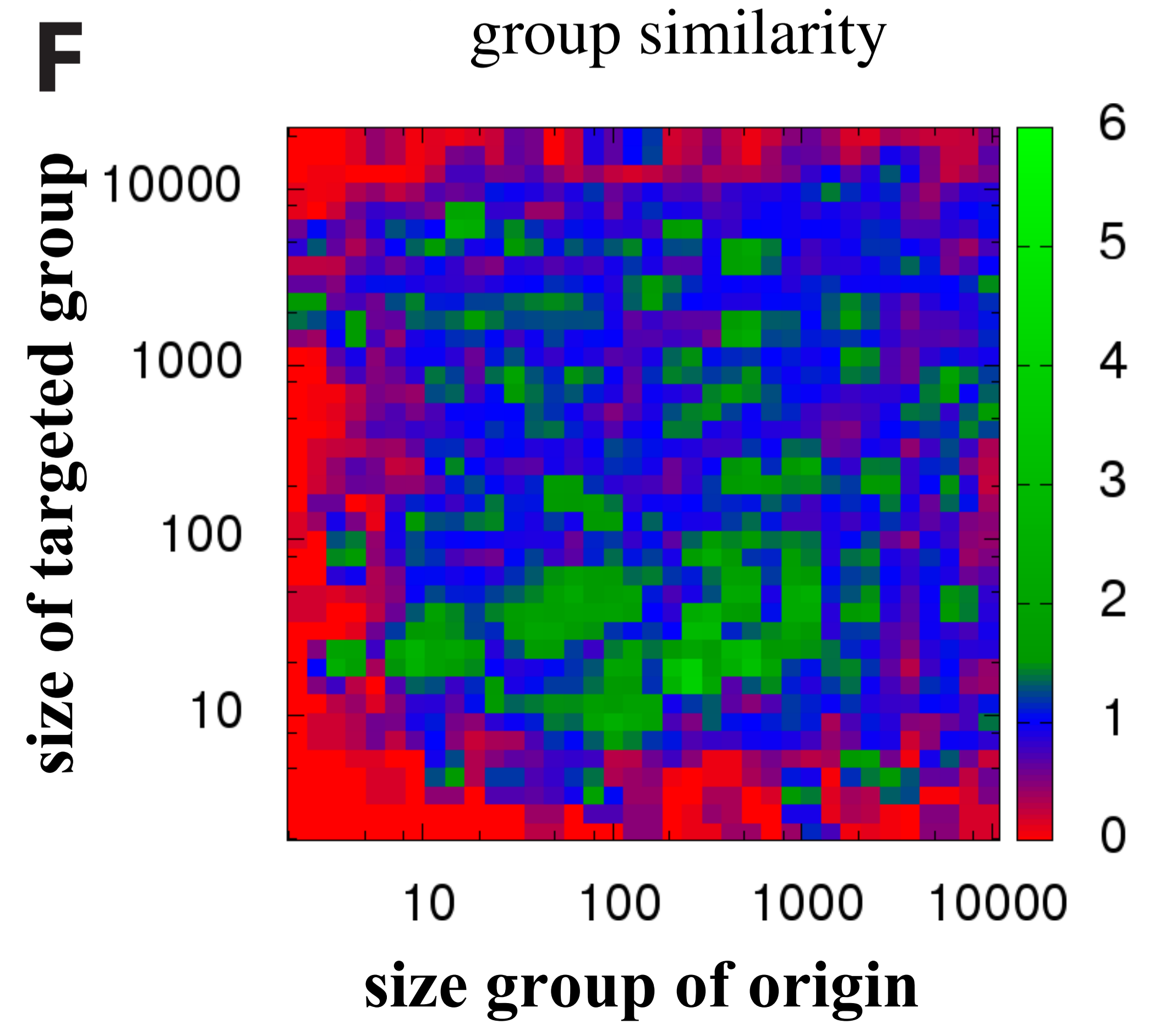

Supplement: Figure S12 — Activity on between-groups links when the groups are detected by Infomap in the sample without hubs. The panel reproduces the structure of Figure 3 of the main paper and of Figure S3. (A) Fraction of links in the follower networks as a function of the size of the group of origin and destination. (B) and (C) Fraction of links of different types: follower relations (black circles), links with mentions (red squares) or with retweets (green diamonds), as a function of the size of the group of origin or destination, respectively. (D) Frequency of links of the different types as a function of the group-group similarity. Ratio between the average group similarity for the links between groups with mentions (E) or retweets (F) and the follower network as function of the size of the group of origin and destination. (PDF) [file pone.0029358.s012.pdf]

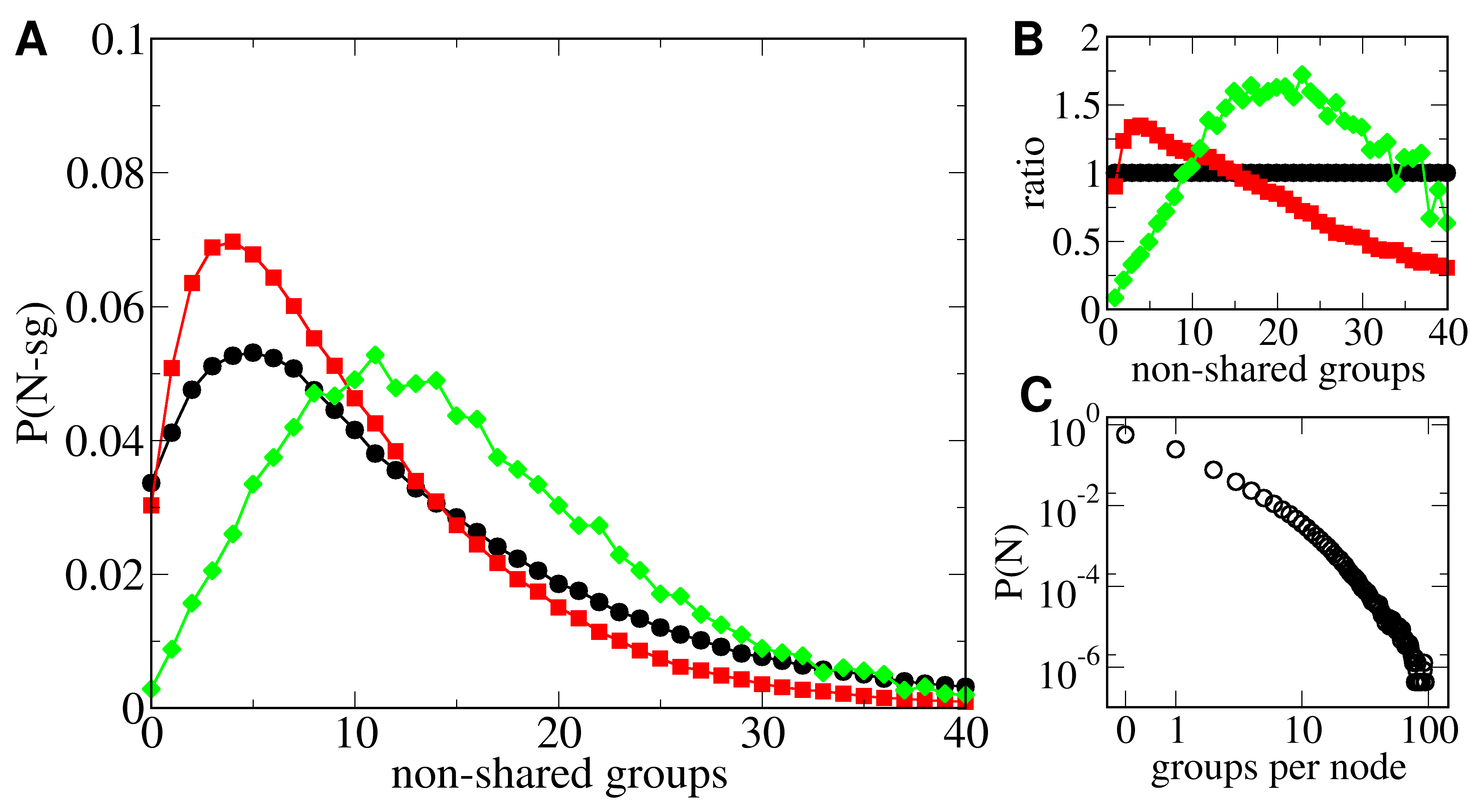

Supplement: Figure S13 — Bridges between groups detected by Moses for the network sample without hubs. (A) Distribution of the links in the follower network (black curve), those with mentions (red curve) and retweets (green curve) as a function of the number of not-shared groups of the users at the extreme of the link. (B) Ratio between these distributions taking the follower network as baseline. (C) Distribution of the number of groups to which each user is assigned. (PNG) [file pone.0029358.s013.png]

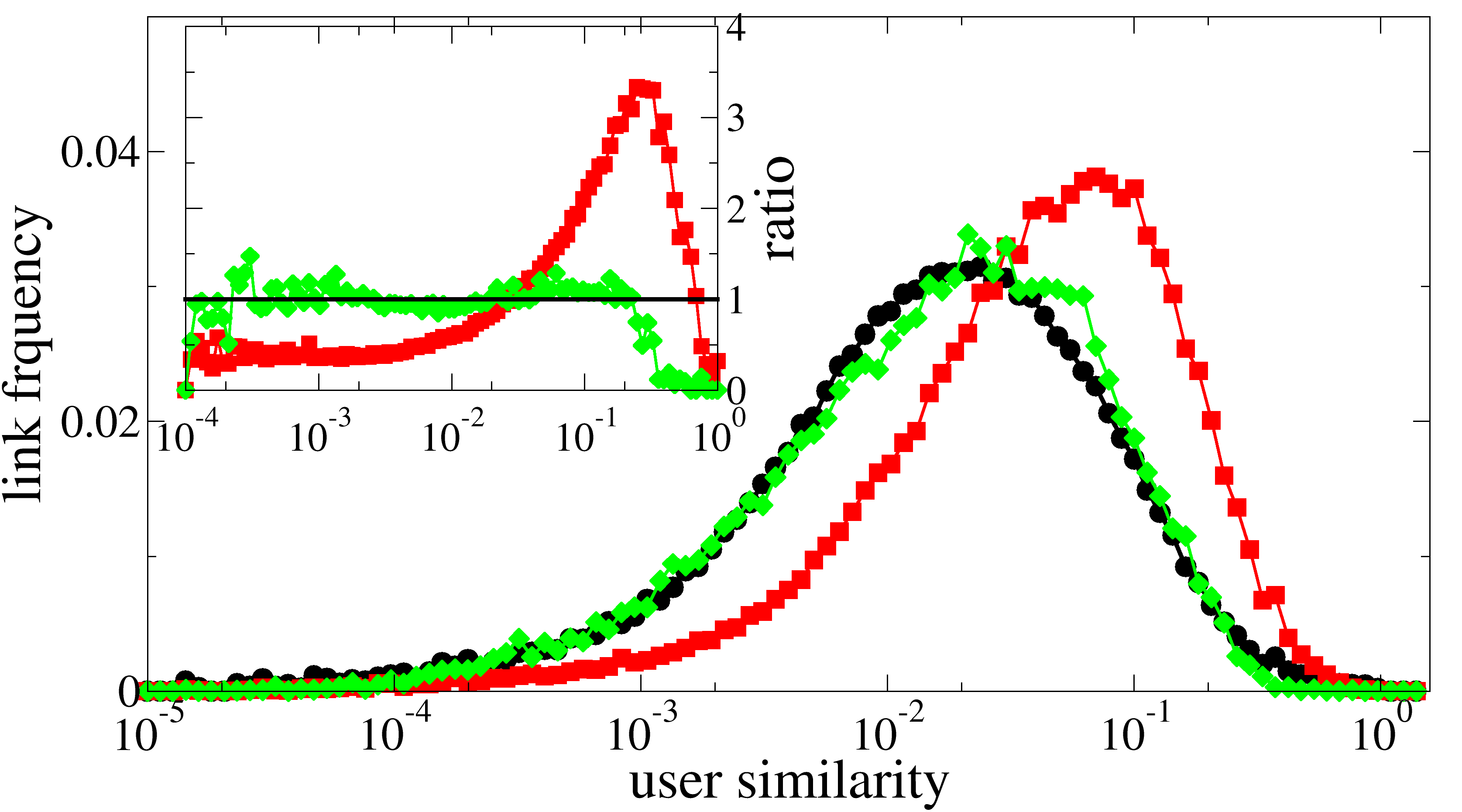

Supplement: Figure S14 — Jaccard similarity of users followers. Users similarity frequency for pairs of users connected by a follower link (black circles), by a link with a mention (red squares) and a link with retweet (green diamonds). Inset: ratio between these frequencies taking the follower network as a baseline. (PNG) [file pone.0029358.s014.png]
