# Supplementary material for: Social Features of Online Networks: The Strength of Intermediary Ties in Online Social Media
Source: PLoS One. 2012 Jan 11;7(1):e29358. doi: 10.1371/journal.pone.0029358 (PMC3256152; doi:10.1371/journal.pone.0029358)
Supplement: Table S1 — Summary of the results regarding internal connections when the groups are obtained with several clustering algorithms for different samples of the network. We measure the trend of the mentions to concentrate in internal connections. Legend: - weak signal, - signal only for small groups, typically smaller than members, a hyphen is inserted if we have no results. (PDF) [file pone.0029358.s015.pdf]

Table 1: Summary of the results regarding internal connections when the groups are obtained with several clustering algorithms for different samples of the network. We measure the trend of the mentions to concentrate in internal connections. Legend: w - weak signal, sg - signal only for small groups, typically smaller than 10 members, a hyphen is inserted if we have no results.

Network sample	Nodes	Edges	Oslom	Infomap	Moses	Real-time	Louvain	Radatools	Figure
Whole network	2 408 534	48 776 888	✔	✔	-	✔	w	-	S6
Snowball 2 hops	61 492	5 558 036	✔	✔	✔	✗	✗	-	S7
Snowball 3 hops	175 078	10 356 020	✔	✔	✔	w	✔	-	S8
Random 200k	200 000	346 578	✔	✔	✔	sg	sg	sg	S9
No hubs	2 395 415	23 404 103	-	✔	✔	w	✗	-	S10
Oslom groups	99 832	1 216 942	✔	✔	✔	✔	✔	✔	S11
